# Identification and Characterization of the Insecticidal Toxin “Makes Caterpillars Floppy” in *Photorhabdus temperata* M1021 Using a Cosmid Library

**DOI:** 10.3390/toxins6072024

**Published:** 2014-07-10

**Authors:** Ihsan Ullah, Eun-Kyung Jang, Min-Sung Kim, Jin-Ho Shin, Gun-Seok Park, Abdur Rahim Khan, Sung-Jun Hong, Byung-Kwon Jung, JungBae Choi, YeongJun Park, Yunyoung Kwak, Jae-Ho Shin

**Affiliations:** 1 School of Applied Biosciences, College of Agriculture and Life Sciences, Kyungpook National University, Daegu 702-701, Korea; E-Mails: ihsanknu@gmail.com (I.U.); mslily87@nate.com (M.-S.K.); kormidas@naver.com (J.-H.S.); parkgun00700@nate.com (G.-S.P.); rahimkhan_84@yahoo.com (A.R.K.); jjanghsj@naver.com (S.-J.H.); bkjung@knu.ac.kr (B.-K.J.); chjba@hanmail.net (J.C.); wofoddl313@naver.com (Y.P.); yun@knu.ac.kr (Y.K.); 2 Food and Biological Resources Examination Division, Korean Intellectual Property Office, Daejeon 302-701, Korea; E-Mail: jangek38@korea.kr

**Keywords:** insecticidal toxin, *Galleria mellonella*, makes caterpillar floppy, *Photorhabdus temperata*, *Tenebrio molitor*

## Abstract

*Photorhabdus temperata* is an entomopathogenic enterobacterium; it is a nematode symbiont that possesses pathogenicity islands involved in insect virulence. Herein, we constructed a *P. temperata* M1021 cosmid library in *Escherichia* coli XL1-Blue MRF` and obtained 7.14 × 10^5^ clones. However, only 1020 physiologically active clones were screened for insect virulence factors by injection of each *E. coli* cosmid clone into *Galleria mellonella* and *Tenebrio molitor* larvae. A single cosmid clone, PtC1015, was consequently selected due to its characteristic virulent properties, e.g., loss of body turgor followed by death of larvae when the clone was injected into the hemocoel. The sequence alignment against the available sequences in Swiss-Prot and NCBI databases, confirmed the presence of the *mcf* gene homolog in the genome of *P. temperata* M1021 showing 85% homology and 98% query coverage with the *P. luminescens* counterpart. Furthermore, a 2932 amino acid long Mcf protein revealed limited similarity with three protein domains. The N-terminus of the Mcf encompassed consensus sequence for a BH3 domain, the central region revealed similarity to toxin B, and the C-terminus of Mcf revealed similarity to the bacterial export domain of ApxIVA, an RTX-like toxin. In short, the Mcf toxin is likely to play a role in the elimination of insect pests, making it a promising model for use in the agricultural field.

## 1. Introduction

The *Photorhabdus* genus consists of nematode symbionts, *i.e.*, entomopathogenic Enterobacteria residing within the gut of an infective julienne nematode (*Heterorhabditis bacteriophora*); these bacteria kill a wide range of insects after infection [1]. In addition, *Photorhabdus* is a member of the Enterobacteriaceae, and it is easy to conduct comparative genomic analysis of putative and actual virulence factors with well-studied model bacteria such as *Escherichia coli* [2]. However, more than 50% of *Photorhabdus* genes in gene pools are distinct from their counterparts in the genome of *E. coli* K12, indicating that the *Photorhabdus* genome may contain a large number of novel genes involved in pathogenicity and symbiosis [2]. Genomic sequencing of *Photorhabdus* bacteria such as *P. temperata* M1021 [3] and *P. luminescens* TT01 [4] has revealed numerous pathogenicity islands scattered throughout their genome. These pathogenicity islands encode different groups of toxins including toxin complexes (Tcs), *Photorhabdus* insect-related (Pir) proteins, *makes caterpillars floppy* (Mcf) toxins, and the *Photorhabdus* virulence cassettes (PVC) [5]. The Tcs have been identified as high molecular weight insecticidal toxins with an estimated molecular weight of approximately 1000 kDa, which have been sub-categorized into four different complexes, including Tca, Tcb, Tcc and Tcd [4]. All the four groups exhibit injectable toxicity against insects, whereas two of the complexes, Tca and Tcd, show oral toxicity as well [6]. In addition, Tca has been revealed to disrupt the epithelial cell line of the insect midgut, in a way similar to the δ-endotoxins from *Bacillus thuringiensis* (Bt) [6]. The “Pir” toxins of the *Photorhabdus* bacteria consist of two well-known members; PirA and PirB, collective known as “PirAB”. The “PirAB” have been shown to be binary toxins with both injectable and oral toxicities towards insects. However, it has been found more effective against the mosquitoes especially against the Dengue vectors, *Aedes aegypti* and *Aedes albopictus* [7]. The PVC toxins have been investigated to be homologous of prophage-like proteins of *S. entomophila*, structurally similar to R-type pyocins [7]. The PVCs cause toxicities against a variety of insects, including *G. mellonella* and *M. sexta*, by destroying the insect hemocytes, which undergo dramatic actin cytoskeleton condensation [8]. Moreover, Mcf is multi-domain toxin and almost all of the *Photorhabdus* species carry *mcf* gene at differing genomic locations, indicating that it is highly mobile within the genome [5]. The Mcf is a potent toxin that is active upon injection and promotes rapid destruction of the insect midgut, resulting in the caterpillars losing all body turgor and becoming “floppy”. In addition, it also destroys the insect phagocytes (hemocytes) by promoting their apoptosis [9]. Furthermore, Mcf promotes toxicity in a variety of insects as well as in human beings [10]. *Photorhabdus* has a complex life cycle, in which it part of the life cycle occurs in the gut of entomopathogenic nematodes as symbiont and another part in the insect body as pathogen [6]. In order to combine symbiosis with the nematode and pathogenicity with the larva of the insect, *Photorhabdus* must produce factors that can do both [7,8]. During the pathogenic phase, the bacteria produce toxins, enzymes, bacteriocins and antibiotics, which cause toxicity in the insects, as well as nutritionalize the insect body [9,10,11,12]. Chitinase has also been identified as cytotoxic enzyme, produced by *Photorhabdus* during the pathogenic phase, which exhibits oral insecticidal activity against insects [13]. In addition, these islands also encode for genes that are responsible for symbiosis and nematode growth [14,15]. These data demonstrate the potential uses of *Photorhabdus* toxins as a substitute of Bt toxins in agriculture [16].

The aim of the present study was to identify virulence factors that are present in the gene pool of *P. temperata* M1021, making it extremely virulent against a wide range of insects. A cosmid library of genomic DNA from *P. temperata* M1021 was constructed in *E. coli* XLI-Blue MRF` and screened for the insect virulence factor(s) by using the injection of an individual clone to combat *Galleria mellonella* and *Tenebrio molitor* larvae. Here, we isolated 1020 physiologically active clones and subsequently selected a single clone “PtC1015” showing the similar virulence characteristics as the Mcf toxin.

## 2. Results and Discussion

### 2.1. Screening of the Cosmid Library

Bioinformatics of the *Photorhabdus* genus revealed pathogenicity islands encoding numerous putative and established toxins [17,18]. Numerous strategies including purification through chromatography and cosmid clone strategy have been adapted to glean highly active toxins from the *Photorhabdus* spp. against the wide range of insects [19]. However, toxin attained through different purification strategies may lose their activities due to alteration in structure, at either end, or it may become protected in some way from inactivation in the insect gut [20]. The cosmid clone approach, however, provides *in vivo* factors that facilitate the mutual interaction of the proteins and keep them active against the host [21].

In order to identify other insect toxins from the bacteria, a cosmid library of *P. temperata* M1021 genomic DNA (pPtC, total 7.14 × 10^5^ clones) was constructed. The biologically active clones were assessed via screening, in which 1020 clones were selected by a bioassay against fully mature (fifth instar) larvae of *G. mellonella* and *T. molitor*. The clones were tested by an intra-hemocoel injection bioassay, a method that delivers the bacteria directly into the hemolymph and thus mimics the release of the bacteria into the hemolymph that occurs soon after a nematode infects the insect host [22,23,24]. The bioassay results indicated that intrahemocoel injection of physiologically active clones killed various percentages of larvae. Through the initial screening, 30 clones were identified with different levels of insecticidal activity, and subsequent screening was confined to 11 clones. The toxicity patterns of the 11 clones were morphologically different from each other in larvae e.g., dark black, black spots, blackish, dark brown, dark red, gray color. In addition, some larvae also exhibited loss of body turgor, resulting in the floppy phenotype (Tables S1 and S2).

**Figure 1:**
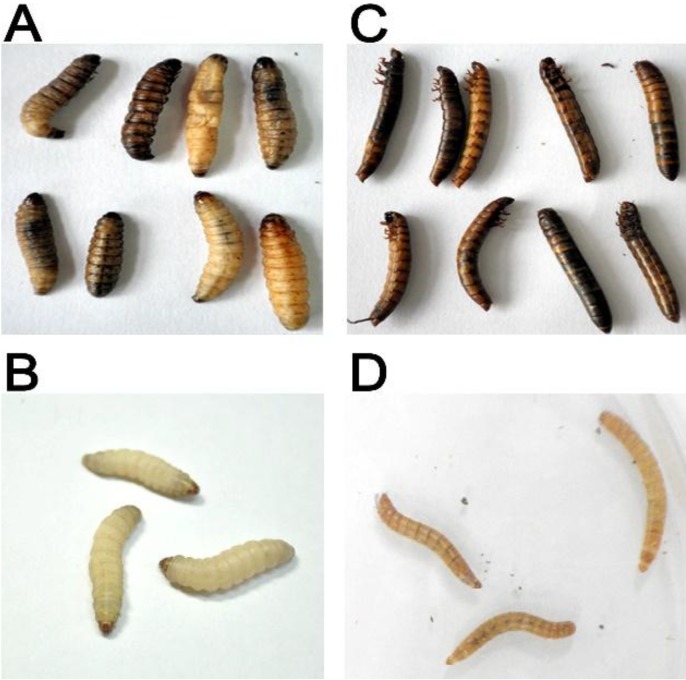
Toxicity pattern of an *E. coli* cosmid clone “PtC1015” against the insect larvae. The clone was bio-assayed via intrahemocoel injection into the larvae that caused death within 24 h. (**A**) The PtC1015 clone was injected into the hemocoel of *G. mellonella* larvae, showed a floppy appearance, indicating “*make caterpillars floppy*” characteristics; (**B**) as control of A, *E. coli* XL1-Blue MRF` harboring plasmid SuperCos1 was injected to *G. mellonella* larva, indicating no insecticidal activity; (**C**) the PtC1015 clone was injected into the hemocoel of *T. molitor* larvae, which caused death within 24 h; (**D**) as control of C, *E. coli* XL1-Blue MRF` harboring plasmid SuperCos1 was injected to *T. molitor* larva, indicating no insecticidal activity.

Finally a single clone, PtC1015, causing the floppy phenotype was selected for further characterization. The intrahemocoel injection of PtC1015 into the *G. mellonella* showed a rapid loss of body turgor, followed by death of *G. mellonella* in reference to larvae injected with control (Figure 1A,B). Similarly, the *T. molitor* larvae were observed to be dead within 24 h after the injection of PtC1015 as compared to larvae injected with control (Figure 1C,D). *E. coli* XL1-Blue MRF` containing non-recombinant SuperCos1 was used as control. It should be noted that the toxins such as Tcs, Pir, PVCs and Mcf produced by *Photorhabdus* are active upon injection. However, one of the noteworthy features of the PtC1015 toxicity was forcing the caterpillar into floppy morphology, which has been a characteristic feature of Mcf toxin. The toxin promotes rapid destruction in the insect midgut, resulting in the loss of body turgor of the caterpillars [11]. In addition, the Mcf has been investigated to destroy the insect phagocytes (hemocytes) by promoting their apoptosis [7]. Similarly, the genomic DNA of *P. luminescens* has been used to construct a genomic DNA library by Daborn *et al.* [11], through which they identified a single cosmid clone, causing the loss of body turgor and eventual larval death. The loss of body turgor, followed by floppy morphology, is due to the destruction of the insect midgut, endocytosis and cytotoxicity caused by multiple conserved domains present in the Mcf toxin [22].

### 2.2. Sequence Alignment and Bioinformatics Analysis of the mcf Gene

A cosmid clone PtC1015, which showed characteristic features (*makes caterpillars floppy*), of the Mcf toxin was selected for further characterizations. From the cosmid clone PtC1015, the cosmid was extracted and designated as pPtC1015. In order to confirm whether this cosmid harbors the gene encoding the Mcf toxin, PCR amplification targeting the *mcf* gene was carried out using an appropriate primer set with either the genomic DNA of *P. temperata* M1021 or the extracted pPtC1015 as a template DNA. The primer sets for amplifying the various toxin genes as *tcc*B, *tcc*, *tcd*, *tcd*A, *mcf* and chitinase gene were designed based on the genomic information of *P. luminescens* genome (Table 1). In the first trail, primers were designed to amplify the entire open reading frame (ORF) of the toxin genes, but failed to amplify, thus suggesting that the sequence homologies between the primers and terminals region of the template DNA were not enough for the PCR annealing reaction. Furthermore, the primers were designed for the most conserved regions, obtained through the gene alignment of the different *Photorhabdus* spp. to amplify only partial fragments of the toxin genes.

**Table 1:** Specific primer sets for amplifying the various toxin genes components.

Names of the Target Genes	Primers	Primer Sequence (5'→3')
Partial *tcc*B gene (0.46 kb)	TccB1 TccB2	GTG ACT CAG CTT TCA ATC GCC AT GAT AGA TCC GCC ACC ATA TCC AG
Partial *tcc* gene locus (0.84 kb)	Tcc1 Tcc2	AAR YTG GCT GAA GAG CAY GTG CAA TAC CCT TAC CTG TGC
Partial *tcd* gene locus (0.77 kb)	Tcd1 Tcd2	AAG ACC GTT TTT CCC GTT ATG AAT A ATC ACC GGA TTG CAC CAC ATG
Partial *tca* gene locus (1.6 kb)	Tca1 Tca2	GGA ATT CAT GCT GAG CTA TTG GCA A GGT CGA CGC GAA TGG TAT AGC GAA T
Partial *tcd*A gene locus (2.2 kb)	TcdA1 TcdA2	ACC GAT GGA TTT CAG TGG CGC T GCG TCG ACT TGG CGA ATG GTG TAG CGA A
Partial chitinase gene (1.6 kb)	ChiA1 ChiA2	GCG AAT TCC ATA TGT TTA AAA CAA TTG TAT CG CTG AAA GTA CAG GTT CTC ATT TAA TTT GCA CGC TTC ACA
Partial *mcf* gene locus (0.87 kb)	Mcf1 Mcf2	CCT GAC GCC GTT GCC CGA TGA CAC AAC AGG GCA CCG GAT TCA GAG ATG

**Figure 2:**
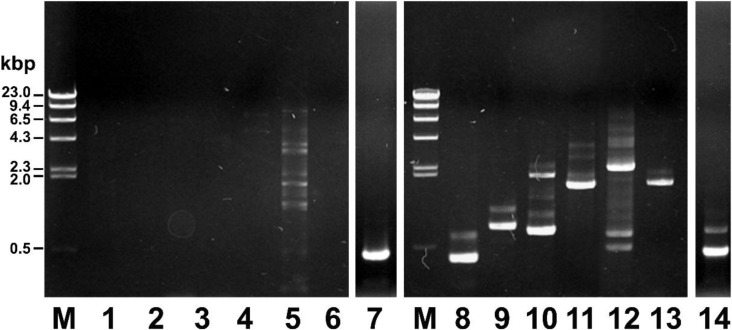
PCR amplification of the *mcf* gene using pPtC1015 as the template DNA. Lanes 1–7, PCR products amplified with the pPtC1015 as a template DNA; lanes 8–14, PCR products amplified with the chromosomal DNA of *P. temperata* M1021 as a template DNA. M: λ DNA digested with *Hind*III marker (0.1 μg each); lanes 1–14, PCR products amplified with specific primer sets targeting for partial *tcc*B gene (lane 1, 8), partial *tcc* gene (lane 2, 9), partial *tcd* gene (lane 3, 10), partial *tca* gene (lane 4, 11), partial *tcd* gene (lane 5, 12), partial chitinase (lane 6, 13) and partial *mcf* gene (lane 7, 14), respectively. Lanes 7 and 14 were taken from a separate gel.

As shown in Figure 2, partial *mcf* gene fragments were amplified through PCR by using pPtC1015 and/or genomic DNA as a template (Figure 2, lane 7 and 14, respectively). However, the toxin complexes or chitinase genes remained unamplified during the PCR reaction, where pPtC1015 was used as template DNA (Figure 2, lanes 1–5 for toxin complexes; lane 6 for chitinase gene). Moreover, partial gene fragments of the toxin complexes or chitinase genes were successfully amplified through PCR reaction in which the genomic DNA of *P. temperata* M1021 was used as a template DNA (Figure 2, lanes 8–13). Some non-specific bands were also amplified during the PCR amplification (lane 5 and lanes 8–14). The above results indicate that the pPtC1015 could be a candidate, bearing the *mcf* gene, responsible for loss of body turgor and floppy morphology in the larvae.

Furthermore, the entire inserted fragment of pPtC1015 was subjected to Sanger sequencing. Through the sequence analysis, an 8.8 kb open reading frame of the *mcf* gene was deduced and sequence was deposited at the NCBI with the accession no. KJ584647. The *mcf* gene showed 85% homology in 98% query coverage to the *P. luminescens* TT01 counterpart (Figure S1). The 8.8 kb *mcf* gene encodes a large predicted polypeptide of 2932 amino acids with a molecular weight of 325 kDa. Through the available databases search [25,26,27] for the Mcf toxins, three conserved domains as well as motifs were revealed.

**Figure 3:**
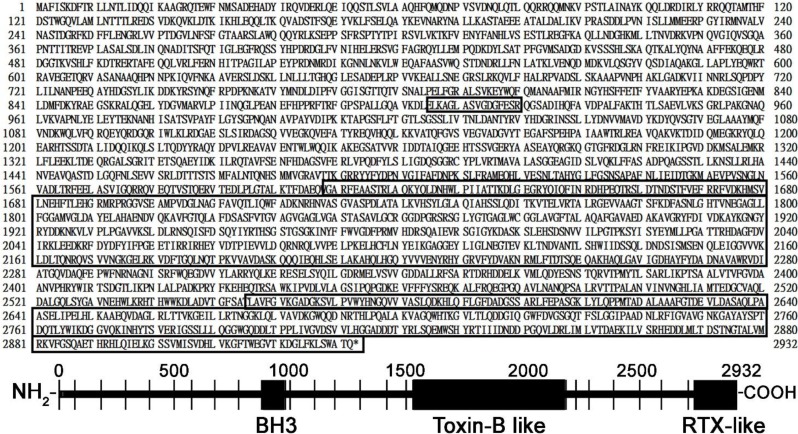
The 2932 amino acid sequence of Mcf toxin with three potential functional domains highlighted in boxes. First box (from 905 to 919) at the N-terminus, carrying a BH3-like consensus domain, suggests the protein may be pro-apoptotic. Second box represents a domain (from 1608 to 2280) has the similarity to the *Clostridium difficile* toxin B (CdtB), responsible for gut destruction. Third box, at the C-terminal (from 2556 to 2932), carries a homolog sequence of ApxIVA from *Actinobacillus pleuropneumoniae*, an RTX-like toxin.

One conserved domain located at the N-terminus of Mcf polypeptide was present between 905 to 919 amino acids, as highlighted in the first box in Figure 3. Consensus sequence for BH3 domains was shown, belonging to anti-apoptotic members of the Bcl-2 family (Figure S2, (I)). According to the study reported by Cheng *et al.* [28], the conserved domain at N-terminus has a core consensus with the BH3 domain, suggesting that Mcf may be mimicking a proapoptotic BH3 domain protein. Furthermore, Kelekar *et al.* [29] explained that the BH3 domains are evolutionarily conserved, but structurally distant proapoptotic members of the Bcl-2 family containing only a short 9–16 stretch of the amino acid consensus sequence; BH3 domains act as early sensors of apoptosis. Previous studies [28,29,30] found that members of this family are highly related in one or more specific regions, collectively called Bcl-2 homology (BH) domains. In addition, Sattler *et al.* [30] demonstrated that the BH domains of the Bcl-2 family contribute their role at multiple levels in cell death and survival.

Another important domain was located at the C terminus, and was 672 amino acids long; it corresponded to the TcdA–TcdB pore-forming region, highlighted in second box of the Figure 3. This conserved region was present between 1608 and 2280 amino acids, bearing up to 21% amino acid similarity to a conserved region of a *C. difficile* toxin-B (CdtB) (Figure S2, (II)). Daborn *et al.* [11] mentioned that the region is putatively involved in the toxin translocation and endocytosis. Hofmann *et al.* [12] pointed out the enzymatic characteristic of the toxin-B, which is a virulent factor of *C. difficile*. In addition, They delineated the mechanism of action and explained that toxin-B covalently modifies low molecular mass GTP-binding proteins of the Rho family by glucosylation, using UDP-glucose as a co-substrate, hence the toxin specifically glucosylates Rho, Rac, and Cdc42 at threonine 37 and threonine 35, respectively, thus inhibiting the bio-activity of the small GTPases [31]. Because Rho proteins are regulators of the actin cytoskeleton, glucosylation results in the destruction of cytoskeleton [12], and the various signal transduction processes that are controlled by GTPases are inhibited by the glucosylation [32]. It is inferred that toxin-B can facilitate the endocytosis and translocation phenomenon, which could be involved in the larval toxicity and floppy formation [11,12].

Finally, C-terminus of Mcf sequence revealed consensus for export domain of ApxIVA, an RTX-like toxin from *A. pleuropneumoniae* (Figure S2, (III)). The similarity was extended to C-terminus from 2556 to 2932 amino acids highlighted in the third box in Figure 3. The RTX family of cytotoxins are related to the pore-forming protein toxins in many Gram-negative pathogens, including *E. coli*, *Bordetella pertussis*, *Pasteurella hemolytica* and *Vibrio cholera* that produce extracellular pore-forming toxins belonging to the RTX (repeats in toxin) family [33,34]. Furthermore, the Mcf from *P. luminescens* was identified as the similar to RtxA toxin which causes the actin cross-linking and depolymerization [11,15,35]. Prochazkova *et al.* [36], elucidated the mechanism of the action of RtxA; RtxA leads the destruction of actin cytoskeleton in the host cells through G-actin modification and destruction of Rho GTPases [37]. The destruction of G-actin in the cytosol of host cell cytosol caused the prevention of actin microfilament formation, a major component of the cytoskeleton, and hence, led to the death of the host [36]. 

**Figure 4:**
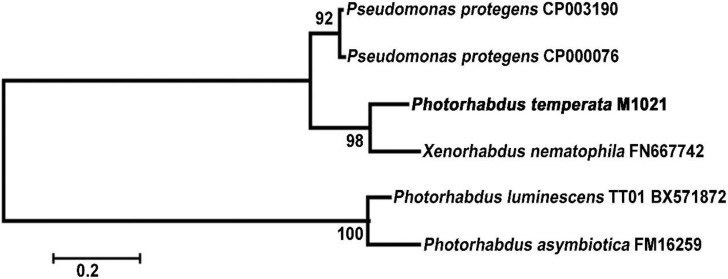
Phylogenetic analysis of the *mcf* gene from *P. temperata* M1021 and other bacteria. BLASTN alignment from the NCBI was used to collect the *mcf* genes from the related strains: *Pseudomonas protegens* CP003190, *P. protegens* CP000076, *X. nematophila*, *P. luminescens* and *P. asymbiotica*. Phylogenetic tree was constructed using the neighbor joining method with 1000 bootstrap replications using the MEGA 4.0 program. Bar indicates the Jukes-Cantor evolutionary distance.

The phylogeny of *mcf* gene was investigated to find the genealogical ties among the *mcf* sequences present in different species of the *Photorhabdus* as well as in other related genera such as *Xenorhabdus* and *Pseudomonas*. As shown in Figure 4, the *mcf* gene from *P. temperata* M1021 was close to the *mcf* counterparts from not only the other *Photorhabdus* bacteria but also the *Pseudomonas* and *Xenorhabdus* bacteria. Interestingly, the *mcf* gene of *P. temperata* was closer to *X. nematophila* which is a member of an entomopathogenic genus other than the *P. luminescens or P. asymbiotica*. Moreover, the *mcf* genes from the two *Pseudomonas* spp. were closely related to the *mcf* gene present in *P. temperata* M1021. According to Wilkinson *et al.* [2], the *mcf* is located in a different part of the genome in the different species of the *Photorhabdus*, thereby adding weight to the assumption that this gene is mobile within the genome. The mobility of the gene could be the reason that *mcf* of *P. temperata* M1021 came closer to *X. nematophila* than *Photorhabdus* spp. 

Results of the present study, e.g., Phylogenetic tree, “floppy” phenotype of the larvae, amino acids sequence homology, and alignment of the putative *mcf* with the sequences present in the NCBI database, indicated that the insecticidal toxicity of the clone PtC1015 can be the activity of the Mcf toxin, reported in different species of *Photorhabdus*, such as *P. luminescens* TT01, *P. luminescens* W14, *P. asymbiotica* and *P. temperata* K1122 [17,18].

### 2.3. Insecticidal Bioassay of the Recombinant Clones against Insect Larvae

**Figure 5:**
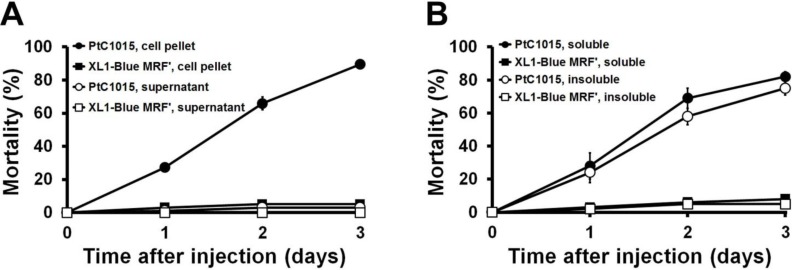
Toxicity of the PtC1015 clone on the *G. mellonella* larvae: (**A**) Mortality caused by the whole cells (1 × 10^3^ CFU), and supernatant (1 μg) injection. Whole cells or supernatant of *E. coli* XL1-Blue MRF` were used as the control. ●, whole cell of PtC1015 clone; ■, whole cell of *E. coli* XL1-Blue MRF`; ○, supernatant from PtC1015 clone; □, supernatant from *E. coli* XL1-Blue MRF`; (**B**) Mortality caused by injection of soluble and insoluble protein fraction (10 μg each). The protein fractions prepared from the *E. coli* XL1-Blue MRF` were used as the control. ●, soluble fraction of PtC1015 clone; ■, soluble fraction of *E. coli* XL1-Blue MRF`; ○, insoluble fraction of PtC1015 clone; □, soluble fraction of *E. coli* XL1-Blue MRF`. All experiments were repeated at least three times and presented with standard deviation. Some error bars (standard deviation) are smaller than the symbols.

The clone PtC1015 containing the *mcf* gene was bio-assayed against to the insect larvae. Three micro-liters of whole cells of the PtC1015 clone (3 × 10^3^ CFU) or 1 μg of its supernatant proteins were injected into the larvae through intrahemocoel injection. The results indicated that up to 90% mortality was caused by whole-cell injections, whilst the mortality rate attributable to the supernatant was insignificant as compared to that of the control (Figure 5A). In addition, soluble and insoluble fractions from the whole-cell lysate were tested using the same injection method; these fractions caused up to 82% and 75% of mortality, respectively (Figure 5B). Rapid death with characteristic loss of body turgor in the larvae was noticed after the injection of soluble, insoluble, and whole-cell injection samples. As reported in previous studies [22,38], the loss of body turgor on the larvae was generally termed as the “floppy” phenotype, a characteristic feature of the Mcf toxin secreted from the *Photorhabdus* bacteria.

In early reports, Daborn *et al.* [11] demonstrated that disruption of the midgut of the larvae causes the loss of body turgor, leads to the floppy phenotype. Furthermore, previous reports suggested that the Mcf protein has conserved domains similar to a region of *C. difficile* toxin-B, the proapoptotic BH3 domain and the RTX-like domain, responsible for endocytosis, apoptosis and destruction of cytoskeleton [12,13,39]. All these reports reinforce the present results, suggesting that Mcf protein is a causative factor of “making caterpillar floppy” characteristic in larvae. 

All of these injection samples, *i.e.*, whole cell, supernatant, and soluble and insoluble fractions were tested for oral toxicity against *G. mellonella* and *T. molitor* larvae: though the condition of larvae was carefully monitored, yet the oral insecticidal activity was insignificant and the mortality rate was negligible (data not shown). Previous reports by French-Constant *et al.* [15] also revealed the same results, suggesting that unlike the cases of Tcs and Pir toxins, the Mcf toxin is only active via injection. All these confirmatory analyses strongly supported the notion that the toxic protein secreted from the PtC1015 clone was the Mcf toxin and caused the floppy phenotype with apoptosis in the insect larvae.

### 2.4. Effects of Proteinase K and Heat Treatments on the Insecticidal Activity of the Toxin Protein

**Figure 6:**
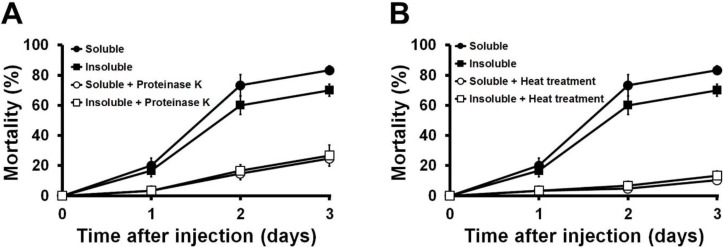
Effect of the heat treatment and proteinase K on the toxicity of protein extracted from PtC1015 clone. (**A**) The mortality rate was affected by the digestion of proteinase K. ●, Soluble fraction without proteinase K digestion; ■, Insoluble fraction without proteinase K digestion; ○, Soluble fraction with proteinase K digestion; □, Insoluble fraction with proteinase K digestion. (**B**) The mortality rate was affected by heat treatment. ●, Soluble fraction without heat treatment; ■, Insoluble fraction without heat treatment; ○, Soluble fraction with heat treatment; □, Insoluble fraction with heat treatment. All experiments were repeated at least three times and values were presented with standard deviation.

The soluble and insoluble protein fractions extracted from the PtC1015 clone, responsible for the insecticidal activities, were subjected to both heat treatment (70 °C for 15 min) and proteinase K digestion (37 °C for 1 h with 10 μg/mL being the final concentration) to characterize the proteinaceous nature of the toxin. The results (Figure 6A,B) indicated that soluble and insoluble injection samples were completely inactivated by both proteinase K and heat treatments. In addition, the mortality rate declined to an insignificant level compared to the control. These results confirmed that the Mcf toxin is exclusively responsible for the toxicity in the larvae and no other impurities were supportive in the insecticidal activity. The toxin protein loses its toxicity owing to the loss of its quaternary and tertiary structures by the application of external heat stress [40]. In addition, proteinase K was used to confirm the status of the toxin protein because it is a reliable method for protein digestion and makes them inactive [41,42]. 

## 3. Experimental Section

### 3.1. Bacterial Culture and Isolation of Genomic DNA

*P. temperata* M1021 was cultured in Luria-Bertani (LB) medium (Sigma, Dorset, UK) at 28 ± 2 °C for 48 h with 180–200 rpm. Bacterial growth was assessed as values of OD_600nm_, using spectrophotometer (Shimadzu, UV-1800, Kyoto, Japan). When the culture reached A_600_ of 1.2–1.5, the genomic DNA was extracted by following procedures: The culture broth of *P. temperata* M1021 was centrifuged at 12,000 × *g* for 15 min, and the pellets were re-suspended in Tris-EDTA buffer (TE buffer: 10 mM Tris-HCl, 1 mM EDTA, pH 8.0). Approximately 10% sodium dodecyl sulfate (SDS) and 0.1 mg/mL proteinase K were added and the mixture was then incubated at 37 °C for 1 h. Subsequently, 0.01% (w/v) of cetyltrimethylammonium bromide (CTAB) solution along with 5 M NaCl (0.7 × volume of total solution, v/v) were added, followed by incubation at 65 °C for 10 min; then, proteins were removed by a common PCI (phenol:chloroform:isoamylalcohol 25:24:1, v/v/v) extraction and 0.6 × volume of isopropanol (v/v) was added to supernatant to precipitate the DNA. The predicated DNA was collected through centrifugation, washed with 70% ethanol and re-suspended in 500 µL of TE buffer. Approximately 5 µL aliquot of the purified DNA was run on 0.8% (w/v) agarose gel set at 100 V for 25 min to confirm the DNA along with standard DNA marker (λ DNA/*Hind* III, 0.02 μg/μL; Sigma-Aldrich, St. Louis, MO, USA).

### 3.2. Construction of the Cosmid Library

Total genomic DNA isolated from *P. temperata* M1021 was meticulously purified through a DNA purification kit (Qiagen, Valencia, CA, USA) and subsequently subjected to the enzymatic digestion for appropriate size selection. The DNA was partially digested using *Sau*3A1 endonuclease (5 U of enzymes; Takara, Ōtsu, Japan) at 37 °C overnight and approximately 35 kb DNA fragments were selected from the digested product. Afterward, the selected DNA fragments with the appropriate size were dephosphorylated and ligated using the T4 DNA ligase (Takara, Ōtsu, Japan) into the SuperCos1 cosmid vector kit (Stratagene Co., La Jolla, CA, USA), according to the procedure followed by Lindler *et al.* [43]. The gene construct was packaged into a λ phage, and allowed to infect the *E. coli* XL1-Blue MRF`. The transduced *E. coli* XL1-Blue MRF` were cultured on the LB agar plates supplemented with 100 μg/mL of ampicillin (Sigma-Aldrich, St. Louis, MO, USA), incubated at 37 °C for 18–20 h. The positive clones were selected according to the procedure described by the manufacturer.

### 3.3. Breeding of Larvae

Wax moth larvae, *G. mellonella* and mealworm larvae, *T. molitor* were reared from their eggs (collected from Daegu, Korea) using artificial media; the larvae maintained their growth in the control environment of the laboratory. The media for *G. mellonella* consisted of wheat bran (600 g), rice bran (600 g), yeast extract (4.5 g), CaCO_3_ (2 g), glycerol (250 mL), water (175 mL), honey (600 mL), and vitamin B complex (600 mg). All the ingredients, except honey and vitamins, were mixed thoroughly and then, the mixture and honey were autoclaved at 121 °C for 15 min in separate containers; subsequently, the mixture, honey, and vitamins were all mixed together. Eggs laid by the wax moth on butter paper were placed in 150 g of the media and then incubated at 28 ± 2 °C with a relative humidity of 50%, until the eggs hatched. The small larvae were then transferred to a larger container containing a larger amount of newly prepared medium and were maintained for the bioassay. Similarly, the *T. molitor* larvae were reared from their eggs in a special chamber at an appropriate temperature and humidity. Eggs of the *T. molitor* on butter papers were put into the moist oatmeal substrate in a sterile container approximately 10" × 17" × 6" and incubated at 25 ± 2 °C with the relative humidity of 50%, whereby the eggs hatched. We kept spraying the container with distilled water once a day to keep the media moist. The hatched larvae were transferred in a large amount of media and stored at 4 °C.

### 3.4. Toxicity Assays of the Recombinant Clones against the Model Larvae

The cosmid library was constructed from the genomic DNA of *P. temperata* M1021 in the *E. coli* XL1-Blue MRF` and assayed against the model larvae of *G. mellonella* and *T. molitor* to assess the toxicity level of the cosmid clones. The individual colonies of *E. coli* XL1-Blue MRF` were cultured in 50 mL of Luria broth, supplemented with 100 µg/mL ampicillin and incubated at 37 °C for 18 ± 2 h with 200 rpm. The culture broth was centrifuged at 12,000 × *g* for 5 min at 4 °C and partitioned into the supernatant and pellets. Supernatant fraction was concentrated up to 50-fold by the ultrafiltration with 10-kDa molecular weight cut-off membranes and applied as injection sample. Furthermore, a small fraction of the pellets was diluted up to 1 × 10^3^ CFU/µL and was then used as whole-cell injection sample. The remaining pellets were re-suspended in 5 mL sterile physiological saline solution (0.85% NaCl) and subjected to sonication. The sonicated sample was centrifuged at 12,000 × *g* at 4 °C for 5 min and a supernatant fraction containing soluble proteins was used as a soluble injection sample. The pellets were washed with 10% SDS to obtain an insoluble suspension, and the fraction was used as an insoluble injection sample. Protein concentration of the injection samples were quantified using Bradford assay [44] and 3 µL of the samples (10 µg of protein) were injected into the hemocoel of fifth instar *G. mellonella* and *T. molitor* larvae by using a 10 μL Hamilton syringe (Hamilton Co, Reno, NV, USA). The injected larvae were transferred into a 90 mm Petri dish and incubated at 25 ± 2 °C with the relative humidity of 50% and the mortality rate of larvae were evaluated for three days. Clones causing insect mortality were screened three times, by injecting 10 individual larvae per clone, in search of a repeatable effect. Larvae injected with positive clones were also examined for marked phenotypic changes, such as color changes *i.e.*, dark black, black spots, blackish, dark brown, dark red and gray color. The larval body conditions, such as loss of body turgor and body stiffness, were also examined during the post-injection incubation of larvae (Tables S1 and S2). The oral toxicity of all the fractions mentioned above were determined in the model larvae by oral administration through artificial diet prepared in the laboratory. Approximately 0.1 mL of each fraction was applied to 1000 mm^3^ of the artificial diet and dried for 15 min under the aseptic environment of the laminar flow. Two larvae each of the *G. mellonella* and *T. molitor* were then put on the each food block and incubated at 25 ± 2 °C for seven days. During the incubation, the percent mortality of larvae was assessed. *E. coli* XL1-Blue MRF` harboring the intact SuperCos1 processed similarly as mentioned previously was used as a negative control for both hemocoel- and oral toxicity assays. Ten larvae were used per treatment and three replicates were assayed and all the experiments were repeated at least three times.

### 3.5. Sequence-Based Analysis of the Selected Cosmid Clone

The clone PtC1015, responsible for the characteristic phenotype (loss of body turgor) in the larvae, was grown in LB broth with 100 μg/mL of ampicillin, incubated at 37 °C for 20 h. The cosmid DNA was extracted using a Qiagen Mini Prep kit (Qiagen, Valencia, CA, USA) and sequenced from both ends with standard primers by Sanger DNA sequencing method (Solgent, Korea).

In addition, for ascertaining which ORF within an individual cosmid was associated with the positive phenotype, PCR-based analysis was conducted to identify and confirm the desired gene responsible for the characteristic toxicity in the larvae. Sets of primers (Table 1) were designed to amplify various loci of the toxin gene(s) and the gene complex. The PCR mixture (50 μL) consisted of 5 μL of 10 × PCR *Pfu* DNA polymerase buffer (200 mM Tris-HCl pH 8.8, 100 mM (NH_4_)_2_SO_4_, 100 mM KCl, 1% Triton X-100, 1 mg/mL BSA, 20 mM MgSO_4_), 2 μL of 2.5 mM dNTP, 1 μL of template DNA (10 ng/μL) and 1 μL each of 10 pmol forward- and reverse primers, 5 μL of dimethyl sulfoxide (DMSO), 1 μL of *Pfu* DNA polymerase (2.5 U/μL), and 37 μL of sterile distilled water. The PCR condition was as following: the reaction consisted of 40 cycles that included 3 min of initial denaturation at 94 °C, 30 s of denaturation at 94 °C, 30 s of annealing at 55 °C, 30 s of elongation at 72 °C, and a final extension for 1 min at 72 °C. PCR products were purified using a PCR purification kit (Qiagen, Valencia, CA, USA) and resolved in a 0.8% (w/v) agarose gel along with a standard marker, λ DNA digested by *Hind*III (0.02 μg/μL in final concentration). The comparison of nucleotide sequences was conducted using the BLASTN program from the National Center for Biotechnology Information (NCBI). The nucleotide sequences were aligned using ClustalW2 from European Bioinformatics Institute (EBI) [45]. Based on the BLASTN alignment, five reference genera, such as *P. protegens* CP003190, *P. protegens* CP000076, *X. nematophila*, *P. luminescens*, and *P. asymbiotica*, *i.e.*, those carrying the *mcf* genes, along with one clone, *P. temperata* M1021, were selected. The phylogenetic tree was constructed using the neighbor-joining method [46] in the MEGA 4.0 program.

### 3.6. Effects of Heat Treatment and Proteinase K Digestion on the Toxin Protein

Thermal inactivation of different forms of the toxin protein (e.g., soluble, insoluble, supernatant, and cell pellet), harvested from PtC1015, were determined by subjecting them to the heat treatment. Each fraction was incubated at 70 °C for 15 min and injected into the hemocoel of larvae. In addition, proteinase K was applied to all the fractions to check if the insecticidal toxicity came from proteins. Ten micrograms per milliliter of proteinase K was added to each fraction and the sample was incubated at 37 °C for 60 min. The treated samples were subsequently injected into the hemocoel of larvae to test the insecticidal activity. To observe the effects of injection samples on larvae, they were placed in a Petri plate and incubated at 25 ± 2 °C with the relative humidity of 50%. The rate of mortality was monitored at 24 h of interval time for 3 days. Ten larvae were used per assay with three replicates and the experiments were performed at least three times.

## 4. Conclusions

The “PtC1015” clone was discovered during screening of a cosmid library generated from the genomic DNA of *P. temperata* M1021. The clone exhibited virulence characteristics, similar to Mcf toxin e.g., the loss of body turgor, followed by death in the larvae. The gene sequencing of the pPtC1015 confirmed the presence of 8.8 kb open reading frame of the *mcf* gene, encoding 325 kDa Mcf toxin. Database searches for the Mcf toxin revealed three areas of similarity with known protein domains. First, Mcf contains a consensus sequence for a BH3 domain and the second conserved region was located between 1608 and 2280 amino acids, bearing up to 21% amino acid homology to a conserved region of CdtB domain. Finally, the predicted C-terminus of Mcf showed sequence similarity to an RTX-like toxin from *A. pleuropneumoniae*. The phylogenetic analysis confirmed that the *mcf* sequences were present in different species of the *Photorhabdus* and *Xenorhabdus*. Toxicity profile of all the active fractions of the PtC1015 indicated the similar virulence characteristic known as “making caterpillar floppy” in larvae.

## Supplementary Files

Supplementary File 1 Supplementary Files (PDF, 3182 KB)
