# Supplementary material for: Identification and Characterization of the Insecticidal Toxin “Makes Caterpillars Floppy” in Photorhabdus temperata M1021 Using a Cosmid Library
Source: Toxins (Basel). 2014 Jul 10;6(7):2024–40. doi: 10.3390/toxins6072024 (PMC4113739; doi:10.3390/toxins6072024)

Supplementary Files

Table S1. First PtC screening, to detect the insecticidal activity.

Series A Batch no.	Plate no.	Mortality of Larvae (Time in Days)			Phenotype of Larvae
		1 day	2 days	3 days	
A5	10	-	-	4/4 *	Black
	11	1/4	1/4	2/4	Black
	13	-	-	2/4	
	18	-	1/4	1/4	
	24	1/4	1/4	4/4	Dark red
	28	-	-	3/4	Black
	29	1/4	2/4	3/4	
A6	1	-	2/4	4/4	Gary
	2	1/4	1/4	2/4	Error
	4	1/4	1/4	2/4	
	6	-	4/4		Black spots
	7	-	1/4	2/4	Black/Black spots
	11	1/4	1/4	2/4	Black
	12	-	1/4	3/4	Dark red
	13	-	2/4	4/4	Dark red
	14	-	-	3/4	Black
	15	1/4	2/4	3/4	Dark red Brown
	16	1/3	3/3		Error
	17	2/3	3/3		Dark brown
	18	1/3	2/3	3/3	
	19	2/3	3/3		Black
	20	2/3	3/3		Black
	21	1/3	3/3		Stiff
	22	-	3/3		Black
	23	-	3/3		Black
	24	-	3/3		Reddish
	25	1/3	2/3	2/3	Black
	26	-	-	-	
	27	-	3/3		Reddish
	28	-	-	-	
	29	1/3	1/3	1/3	
	30	-	-	-	
	31	-	-	-	
	32	-	2/3	3/3	Error
	33	-	-	-	
	34	-	-	-	
	35	-	-	-	
	36	1/3	2/3	2/3	
	37	-	-	2/3	
	38	-	-	-	
	39	-	-	1/3	
	40	1/3	1/3	2/3	Black
	41	-	-	-	
	42	2/3	3/3		Black
	43	2/4	4/4		Black

Table S1. *Cont.*

Series A Batch no.	Plate no.	Mortality of Larvae (Time in Days)			Phenotype of Larvae
		1 day	2 days	3 days	
	1	-	3/3		Black
	2	1/3	1/3	3/3	Black
	3	-	3/3		Black
	4	1/3	2/3	2/3	Black, Black spots
	5	-	3/3		Black
	6	2/3	3/3		
	7	-	3/3		Black
	8	-	3/3		Black, Error
	9	-	3/3		Black spots
	10	3/3			Black spots
	11	2/3	2/3	3/3	
	12	-	-	-	
	13	-	-	-	
	14	-	-	-	
	15	-	-	-	
	16	-	-	-	
	17	-	-	-	
	18	2/3	2/3	2/3	
	19	-	-	-	
	20	1/3	1/3	1/3	Dark red
	21	-	-	-	
	22	-	-	1/3	
	23	1/3	1/3	1/3	Dark red
A7	24	1/3	1/3	1/3	Dark red
	25	-	-	-	
	26	1/3	1/3	3/3	Black
	27	-	-	3/3	
	28	1/3	1/3	1/3	
	29	-	-	-	
	30	-	-	-	
	31	-	-	-	
	32	1/3	1/3	1/3	
	33	-	-	-	
	34	-	-	-	
	35	-	1/3	1/3	
	36	-	-	-	
	37	-	-	-	
	38	1/3	1/3	1/3	
	39	1/3	1/3	1/3	Black
	40	2/3	2/3	3/3	black
	41	-	2/3	3/3	Error
	42	-	1/3	2/3	Error
	43	1/3	2/3	2/3	Black
	44	-	1/3	1/3	Black
	45	-	-	-	
	46	1/3	2/3	2/3	Black

Table S1. *Cont.*

Series A Batch no.	Plate no.	Mortality of Larvae (Time in Days)			Phenotype of Larvae
		1 day	2 days	3 days	
	1	-	1/3	1/3	Black
	2	1/3	2/3	3/3	Black, black spots
	3	-	-	2/3	Black
	4	3/3			Dark red
	5	1/3	2/3	2/3	Black
	6	-	2/3	2/3	
	7	-	-	3/3	Dark red
	8	2/3	3/3		Error
	9	2/3	3/3		Black
	10	1/3	3/3		Black
	11	1/3	2/3	2/3	Black
	12	-	1/3	1/3	
	13	-	-	-	
	14	-	-	-	
	15	-	-	-	
	16	-	-	-	
	17	-	3/3		Dark red
	18	-	-	-	
	19	-	-	-	
	20	-	-	-	
	21	-	-	-	
	22	-	1/3	1/3	Black
A8	23	-	-	-	
	24	-	1/3	1/3	Black
	25	-	-	-	
	26	-	-	-	
	27	-	-	1/3	
	28	-	-	-	
	29	-	-	1/3	
	30	-	-	-	
	31	-	-	-	
	32	-	3/3		Stiff
	33	1/3	3/3		Black spots
	34	-	-	-	
	35	-	3/3		Dark red
	36	-	3/3		
	37	-	3/3		
	38	1/3	1/3	1/3	Error
	39	1/3	3/3		Dark red
	40	-	3/3		
	41	1/3	3/3		stiff
	42	-	3/3		
	43	-	3/3		
	44	-	3/3		
	45	3/3			Black spots

Table S1. *Cont.*

Series A Batch no.	Plate no.	Mortality of Larvae (Time in Days)			Phenotype of Larvae
		1 day	2 days	3 days	
A9	1	-	2/3		
	2	-	2/3		
	3	2/3	3/3		Stiff
	4	-	3/3		Stiff
	5	-	2/3		
	6	-	3/3		Stiff
	7	-	2/3		Reddish
	8	-	3/3		Stiff
	9	-	-	-	Black
	10	-	3/3		Stiff
	11	-	2/3	3/3	Stiff
	12	-	1/3	1/3	Dark red
	13	-	1/3	2/3	
	14	-	1/3	1/3	
	15	-	-	-	
	16	-	2/3	2/3	
	17	-	-	-	
	18	-	-	-	Stiff
	19	-	1/3	1/3	
	20	-	1/3	1/3	
	21	-	-	-	
	22	-	2/3	2/3	Dark red
	23	-	2/3	2/3	
	24	-	-	1/3	Black
	25	-	-	1/3	Dark red
	26	-	2/3	2/3	Dark red
	27	-	1/3	1/3	Dark red
	28	-	1/3	1/3	
	29	1/3	2/3	3/3	Stiff
	30	-	1/3	3/3	Dark red, Black
	31	-	1/3	3/3	Black
	32	-	3/3	3/3	Black
	33	-	1/3	3/3	Black
	34	-	-	3/3	Black
	35	-	3/3	3/3	Dark red
	36	-	-	3/3	Black
	37	-	1/3	3/3	Black
	38	-	-	1/3	Black
	39	1/3	1/3	2/3	Black
	40	2/3	2/3	3/3	Black

Table S1. *Cont.*

Series A Batch no.	Plate no.	Mortality of Larvae (Time in Days)			Phenotype of Larvae
		1 day	2 days	3 days	
	1	1/3	1/3	1/3	
	2	1/3	2/3	2/3	
	3	1/3	1/3	1/3	
	4	0/3	0/3	0/3	
	5	0/3	0/3	0/3	
	6	0/3	0/3	1/3	
	7	0/3	0/3	0/3	
	8	0/3	0/3	1/3	
	9	0/3	3/3	3/3	
	10	0/3	3/3	3/3	
	11	0/3	3/3	3/3	stiff
	12	0/3	3/3	3/3	
	13	0/3	3/3	3/3	
	14	0/3	3/3	3/3	Stiff
	15	0/3	3/3	3/3	Stiff
	16	0/3	3/3	3/3	Error
	17	0/3	3/3	3/3	
	18	0/3	3/3	3/3	
	19	0/3	3/3	3/3	
	20	0/3	3/3	3/3	
	21	0/3	3/3	3/3	
	22	0/3	3/3	3/3	
	23	0/3	3/3	3/3	
A10	24	0/3	3/3	3/3	Error
	25	0/3	3/3	3/3	
	26	0/3	3/3	3/3	
	27	0/3	3/3	3/3	Error
	28	0/3	3/3	3/3	
	29	0/3	0/3	0/3	
	30	0/3	0/3	0/3	
	31	0/3	0/3	0/3	
	32	0/3	1/3	1/3	
	33	0/3	0/3	0/3	
	34	0/3	0/3	1/3	
	35	0/3	1/3	1/3	
	36	0/3	0/3	0/3	
	37	0/3	1/3	1/3	Stiff
	38	1/3	1/3	1/3	Stiff
	39	0/3	0/3	0/3	
	40	0/3	0/3	0/3	
	41	0/3	0/3	0/3	
	42	0/3	0/3	0/3	
	43	0/3	0/3	0/3	
	44	1/3	1/3	1/3	
	45	0/3	0/3	0/3	
	46	0/3	0/3	0/3	
	47	0/3	0/3	0/3	

Table S1. *Cont.*

Series B Batch no.	Plate no.	Mortality of Larvae (Time in Days)			Phenotype of Larvae
		1 day	2 days	3 days	
B1	1	0/3	0/3	0/3	
	2	0/3	1/3	1/3	Stiff
	3	0/3	1/3	1/3	Stiff
	4	0/3	1/3	1/3	Stiff
	5	0/3	0/3	0/3	
	6	0/3	0/3	0/3	
	7	0/3	0/3	0/3	
	8	0/3	0/3	0/3	
	9	0/3	0/3	0/3	
	10	0/3	0/3	0/3	
	11	0/3	1/3	0/3	
	12	0/3	0/3	0/3	
	13	0/3	0/3	0/3	
	14	0/3	0/3	0/3	
	15	0/3	1/3	1/3	Error
	16	0/3	1/3	1/3	
	17	0/3	1/3	1/3	
	18	0/3	0/3	1/3	Stiff
	19	0/3	1/3	1/3	Error
	20	0/3	0/3	0/3	
B2	1	0/3	0/3	0/3	
	2	0/3	1/3	1/3	
	3	0/3	1/3	1/3	Error
	4	1/3	1/3	1/3	Stiff
	5	0/3	0/3	0/3	
	6	1/3	1/3	1/3	
	7	1/3	2/3	2/3	
	8	1/3	2/3	2/3	Stiff
	9	1/3	1/3	1/3	Stiff
	10	0/3	1/3	1/3	
	11	0/3	0/3	0/3	
	12	0/3	0/3	0/3	
	13	0/3	0/3	0/3	
	14	0/3	0/3	0/3	
	15	0/3	0/3	0/3	
	16	0/3	0/3	0/3	
	17	1/3	2/3	2/3	
	18	1/3	2/3	2/3	
	19	0/3	0/3	0/3	
	20	0/3	1/3	1/3	
	21	1/3	2/3	2/3	
	22	0/3	0/3	1/3	
	23	0/3	0/3	0/3	
	24	1/3	1/3	1/3	
	25	1/3	1/3	2/3	

Table S1. *Cont.*

Series B Batch no.	Plate no.	Mortality of Larvae (Time in Days)			Phenotype of Larvae
		1 day	2 days	3 days	
B2	26	0/3	0/3	1/3	
	27	0/3	0/3	0/3	
	28	1/3	1/3	1/3	
	29	0/3	0/3	0/3	
	30	0/3	1/3	1/3	
	31	0/3	1/3	1/3	
	32	1/3	1/3	1/3	
	33	0/3	0/3	0/3	
	34	0/3	0/3	0/3	
	35	1/3	1/3	1/3	Stiff
	36	1/3	1/3	1/3	Black
	37	0/3	0/3	0/3	
	38	0/3	0/3	0/3	
	39	0/3	0/3	0/3	
	40	0/3	0/3	0/3	
	41	0/3	0/3	0/3	
	42	0/3	0/3	0/3	
	43	0/3	0/3	0/3	
	44	0/3	0/3	0/3	
	45	0/3	0/3	0/3	
	46	0/3	0/3	0/3	
	47	0/3	0/3	0/3	

* Death/Total.

Table 2. Second round of PtC sc.eening to detect the insecticidal activity.

Series A Batch no.	Plate no.	Mortality of Larvae (Time in Days)			Phenotype of Larvae
		1 day	2 days	3 days	
A5	10	-	-	4/4	Black
	11	1/4	1/4	2/4	error
	13	-	-	2/4	
	18	-	1/4	1/4	
	24	1/4	1/4	4/4	Black
	28	-	-	3/4	Black
	29	1/4	2/4	3/4	
A6	11	-	2/4	4/4	Gray
	13	1/4	1/4	4/4	Error
	20	1/4	1/4	2/4	
	21		4/4		Black
	28	-	1/4	2/4	Black
	29	1/4	1/4	2/4	Black
	39	-	1/4	3/4	Dark red
	45	-	2/4	4/4	Dark red
	48	-	-	3/4	Black

Table S2. *Cont.*

Series A Batch no.	Plate no.	Mortality of Larvae (Time in Days)			Phenotype of Larvae
		1 day	2 days	3 days	
A7	3	3/3	3/3	3/3	Brown
	5	2/3	2/3	3/3	Error
	11	-	-	-	Dark red
	15	-	-	-	
	16	-	-	-	Dark red
	18	-	-	-	Black
	19	-	-	-	Black
	22	2/3	2/3	2/3	Black
	26	-	-	-	Black
	27	1/3	1/3	1/3	Dark red
	38	-	-	-	Black
	39	-	-	1/3	
A9	2	-	3/3	-	Dark red
	5	-	-	-	
	8	1/3	1/3	1/3	
	16	-	-	-	
	19	-	-	-	
	20	-	2/3	3/3	Error
	21	-	-	-	
	30	-	-	-	
	37	-	-		
	41	1/3	2/3	2/3	
A10	30	-	1/3	3/3	Dark red
	31	-	1/3	3/3	Black
	32	-	3/3	3/3	Black
	33	-	1/3	3/3	Black
	34	-	-	3/3	Black
	35	-	3/3	3/3	Dark red
	36	-	-	3/3	Black
	37	-	1/3	3/3	Black
	38	-	-	1/3	Black
	39	1/3	1/3	2/3	Black
	40	2/3	2/3	3/3	Black

Table S2. *Cont.*

Series B Batch no.	Plate no.	Mortality of Larvae (Time in Days)			Phenotype of Larvae
		1 day	2 day	3 day	
B1	2	1/3	1/3	1/3	Stiff
	3	1/3	2/3	2/3	
	5	1/3	1/3	1/3	
	7	-	-	-	
	8	-	-	-	
	10	-	-	1/3	
	12	-	-	-	
	15	-	-	1/3	
	20	-	3/3	3/3	
	21	-	3/3	3/3	
	26	-	3/3	3/3	
	29	-	3/3	3/3	
	36	-	3/3	3/3	
	38	-	-	1/3	
	40	-	1/3	1/3	
	41	-	-	-	
B2	1	-	-	2/3	
	2	-	-	-	
	3	-	-	1/3	Black
	9	1/3	1/3	2/3	Dark red
	10	-	-	-	
	29	2/3	3/3		Black
	30	2/3	3/3	3/3	Dark red
	32	1/3	1/3	1/3	
	35	-	-	-	
	38	-	-	-	
	42	-	-	-	Black

Note: The dash (-) mark represents that the injection cause no mortality in the larvae.

Figure S1. The putative toxin gene sequence from the *Photorhabdus temperata* M1021 was aligned against the *mcf* gene of *Photorhabdus luminescens* TT01 using a CLC genomics work bench version 7.0 alignment tool. The alignment revealed 85% of the sequence similarity and 98% of the query coverage, indicating that the gene isolated and sequenced from *P. temperata* M1021 was the *mcf* gene, encoding "*makes caterpillars floppy*" (Mcf) toxin.

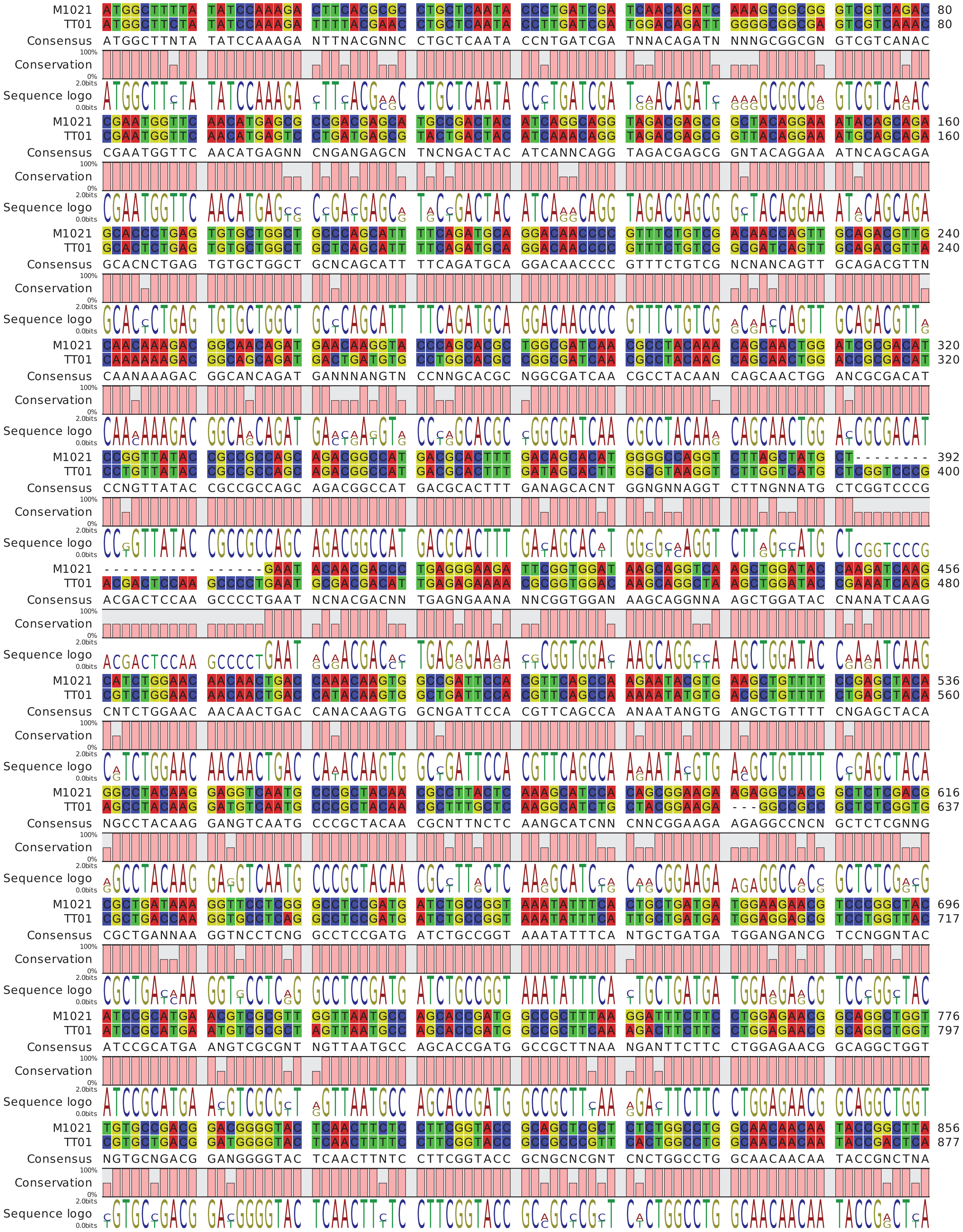
M1021 ATGGCTTTTA TATCCAAAGA CTTCACGCGC CTGCTCAATA CCCTGATCGA TCAACAGATC AAAGCGGCGG GTCGTCAGAC 80
TT01 ATGGCTTCTA TATCCAAAGA TTTTACGAAC CTGCTCAATA CCTTGATCGA TGGACAGATT GGGGCGGCGA GTCGTCAAAC 80
Consensus ATGGCTTNTA TATCCAAAGA NTTNACGNNC CTGCTCAATA CCNTGATCGA TNNACAGATN NNNGCGGCGN GTCGTCANAC
Conservation
100%
0%
Sequence logo
2.0bits
0.0bits
M1021 CGAATGGTTC AACATGAGCG CCGACGAGCA TGCCGACTAC ATCAGGCAGG TAGACGAGCG GCTACAGGAA ATACAGCAGA 160
TT01 CGAATGGTTC AACATGAGTC CTGATGAGCG TACTGACTAC ATCAAACAGG TAGACGAGCG GTTACAGGAA ATGCAGCAGA 160
Consensus CGAATGGTTC AACATGAGNN CNGANGAGCN TNCNGACTAC ATCANNCAGG TAGACGAGCG GNTACAGGAA ATNCAGCAGA
M1021 GCACCCTGAG TGTGCTGGCT GCCCAGCATT TTCAGATGCA GGACAACCCC GTTTCTGTCG ACAACCAGTT GCAGACGTTG 240
TT01 GCACTCTGAG TGTGCTGGCT GCTCAGCATT TTCAGATGCA GGACAACCCC GTTTCTGTCG GCGATCAGTT GCAGACGTTA 240
Consensus GCACNCTGAG TGTGCTGGCT GCNCAGCATT TTCAGATGCA GGACAACCCC GTTTCTGTCG NCNANCAGTT GCAGACGTTN
M1021 CAACAAAGAC GGCAACAGAT GAACAAGGTA CCCAGCACGC TGGCGATCAA CGCCTACAAA CAGCAACTGG ATCGCGACAT 320
TT01 CAAAAAAGAC GGCAGCAGAT GACTGATGTG CCTGGCACGC CGGCGATCAA CGCCTACAAG CAGCAACTGG ACCGCGACAT 320
Consensus CAANAAAGAC GGCANCAGAT GANNNANGTN CCNNGCACGC NGGCGATCAA CGCCTACAAN CAGCAACTGG ANCGCGACAT
M1021 CCGGTTATAC CGCCGCCAGC AGACGGCCAT GACGCACTTT GACAGCACAT GGGGCCAGGT CTTAGCTATG CT-------- 392
TT01 CCTGTTATAC CGCCGCCAGC AGACGGCCAT GACGCACTTT GATAGCACTT GGCGTAAGGT CTTGGTCATG CTCGGTCCCG 400
Consensus CCNGTTATAC CGCCGCCAGC AGACGGCCAT GACGCACTTT GANAGCACNT GGNGNNAGGT CTTNGNNATG CTCGGTCCCG
M1021 ---------- ------GAAT ACAACGACCC TGAGGGAAGA TTCGGTGGAT AAGCAGGTCA AGCTGGATAC CAAGATCAAG 456
TT01 ACGACTCCAA GCCCCTGAAT GCGACGACAT TGAGAGAAAA CGCGGTGGAC AAGCAGGCTA AGCTGGATAC CGAAATCAAG 480
Consensus ACGACTCCAA GCCCCTGAAT NCNACGACNN TGAGNGAANA NNCGGTGGAN AAGCAGGNNA AGCTGGATAC CNANATCAAG
M1021 CATCTGGAAC AACAACTGAC CAAACAAGTG GCCGATTCCA CGTTCAGCCA AGAATACGTG AAGCTGTTTT CCGAGCTACA 536
TT01 CGTCTGGAAC AACAACTGAC CATACAAGTG GCTGATTCCA CGTTCAGCCA AAAATATGTG ACGCTGTTTT CTGAGCTACA 560
Consensus CNTCTGGAAC AACAACTGAC CANACAAGTG GCNGATTCCA CGTTCAGCCA ANAATANGTG ANGCTGTTTT CNGAGCTACA
M1021 GGCCTACAAG GAGGTCAATG CCCGCTACAA CGCCTTACTC AAAGCATCCA CAGCGGAAGA AGAGGCCACG GCTCTCGACG 616
TT01 AGCCTACAAG GATGTCAATG CCCGCTACAA CGCTTTGCTC AAGGCATCTG CTACGGAAGA ---GGCCGCC GCTCTCGGTG 637
Consensus NGCCTACAAG GANGTCAATG CCCGCTACAA CGCNTTNCTC AANGCATCNN CNNCGGAAGA AGAGGCCNCN GCTCTCGNNG
M1021 CGCTGATAAA GGTTCCTCGG GCCTCCGATG ATCTGCCGGT AAATATTTCA CTGCTGATGA TGGAAGAACG TCCCGGCTAC 696
TT01 CGCTGACCAA GGTGCCTCAG GCCTCCGATG ATCTGCCGGT AAATATTTCA TTGCTGATGA TGGAGGAGCG TCCTGGTTAC 717
Consensus CGCTGANNAA GGTNCCTCNG GCCTCCGATG ATCTGCCGGT AAATATTTCA NTGCTGATGA TGGANGANCG TCCNGGNTAC
M1021 ATCCGCATGA ACGTCGCGTT GGTTAATGCC AGCACCGATG GCCGCTTTAA GGATTTCTTC CTGGAGAACG GCAGGCTGGT 776
TT01 ATCCGCATGA ATGTCGCGCT AGTTAATGCC AGCACCGATG GCCGCTTCAA AGACTTCTTC CTGGAGAACG GCAGGCTGGT 797
Consensus ATCCGCATGA ANGTCGCGNT NGTTAATGCC AGCACCGATG GCCGCTTNAA NGANTTCTTC CTGGAGAACG GCAGGCTGGT
M1021 TGTGCCGACG GACGGGGTAC TCAACTTCTC CTTCGGTACC GCAGCTCGCT CTCTGGCCTG GCAACAACAA TACCGGCTTA 856
TT01 CGTGCTGACG GATGGGGTAC TCAACTTTTC CTTCGGTACC GCCGCCCGTT CACTGGCCTG GCAACAACAA TACCGACTCA 877
Consensus NGTGCNGACG GANGGGGTAC TCAACTTNTC CTTCGGTACC GCNGCNCGNT CNCTGGCCTG GCAACAACAA TACCGNCTNA

```
M1021      AGAGTGAGCC GCCATCCTTT CGCTCGCCGA CCTATACGCC AATTCGCTCG GTGCTGGTCA AAACCAAATT CGTCGAAAAT  936
TT01       AGAGTGAGCC GCCATCTTTT CGCTCGCCGA CTTATACGCC AATTCGCTCG GTGCTGGTCA AAACCGAATT CGTCGAGAAG  957
Consensus  AGAGTGAGCC GCCATCNTTT CGCTCGCCGA CNTATACGCC AATTCGCTCG GTGCTGGTCA AAACCNAATT CGTCGANAAN

M1021      TACTTCGCCA ACCACCTGGT TTCTGAAAGC ACCTTGCGCG AAGGTTTCAA GGCGCAGTTG CTGAATGACG GTCACAAGAT  1016
TT01       TACTTCGCCA ACTACCTGGT TTCAGAAAGC ACCTTGCGCG GAGGTTTCAA GGCTCAGTTG CTGGGTAACG GTCGCAAGAT  1037
Consensus  TACTTCGCCA ACNACCTGGT TTCNGAAAGC ACCTTGCGCG NAGGTTTCAA GGCNCAGTTG CTGNNTNACG GTCNCAAGAT

M1021      GCTGCTGACC AACGTTGACC GCAAGGTGCC GAACCAGGTT GGGATTCAAG TGTCCGGCCA AGCTCCGAAT ACTACCATCA  1096
TT01       GCTACTGACT AGCGTTGACC GCAAGGTGCC GAATCAGATC GGGATTCAGG TGTCCGGCCA GGCTCCGAAT ACTACCATCA  1117
Consensus  GCTNCTGACN ANCGTTGACC GCAAGGTGCC GAANCAGNTN GGGATTCANG TGTCCGGCCA NGCTCCGAAT ACTACCATCA

M1021      CCCGTGAGGT GCCATTGGCA AGTGCCTTGA GTGACCTGAT CAACCAGAAT GCTGATATCA CCAGCTTCCA GACTATCGGC  1176
TT01       CCCGTGAAGT GCCATTGGCA AGCGCTTTGA GTGACCTGAT CAACCAGAAT GCTGATATCG CTAGCTTTCG GACTATCGGC  1197
Consensus  CCCGTGANGT GCCATTGGCA AGNGCNTTGA GTGACCTGAT CAACCAGAAT GCTGATATCN CNAGCTTNCN GACTATCGGC

M1021      CTGGAGGGAT TTCGCCAGAG CAGTTACCAC CCAGACCGTG ACGGGCTTTT CGTCAATATC CATGAGCTGG AACGCTCGGT  1256
TT01       TTGGAGGGGT TTCGCCAGAG TAGTTACCAT CCGGACCGTG ACGGGCTTTT CGTCAATATC CATGAGCTGG AACGGTCGGT  1277
Consensus  NTGGAGGGNT TTCGCCAGAG NAGTTACCAN CCNGACCGTG ACGGGCTTTT CGTCAATATC CATGAGCTGG AACGNTCGGT

M1021      CGGTTTTGCC GGTCGCCAGT ACTTGCTGGA AATGCCTCAG GACAAAGACT ACCTGTCCGC GACGCCCTTT GGGGTGATGA  1336
TT01       AGGTTTTGCC GGTCGTCAGT ACTTGCTGGA AATGCCTCAG GACAACGACT ACCTGTCCGC GACGCCTTTT GGGGTGATGA  1357
Consensus  NGGTTTTGCC GGTCGNCAGT ACTTGCTGGA AATGCCTCAG GACAANGACT ACCTGTCCGC GACGCCNTTT GGGGTGATGA

M1021      GCGCTGACGG CGACAAGGTC AGCAGCAGTC ACTTGAGCAA GGCGCAGACC AAAGCACTGT ATCAATACAA CGCTGCATTT  1416
TT01       GCGTTGATGG CGACAAGGTT AGTAGTAGTC ACTTGAGCAA GGCACAGACT GACACACTGT ATCAATACAA CGCCGCATTT  1437
Consensus  GCGNTGANGG CGACAAGGTN AGNAGNAGTC ACTTGAGCAA GGCNCAGACN NANNCACTGT ATCAATACAA CGCNGCATTT

M1021      TTCGAGAAGC AGGAACAACT GCGTGACGGC GGTACGAAGG TCAGTCACCT GTTCAAAGAC ACTCGCGAGC GCACGGCGTT  1496
TT01       TTCGAGAAGT TGGAACAACT GCGTAGCGGC GGTATGAAGG CCAGTCGCTT GTTTGAAGGC AGCATCGAGC GTACAGCGTT  1517
Consensus  TTCGAGAAGN NGGAACAACT GCGTNNCGGC GGTANGAAGG NCAGTCNCNT GTTNNAAGNC ANNNNCGAGC GNACNGCGTT

M1021      CGAGCAGCAG CTGGTACGTT TGTTCGAACG CAACCACATC ACCCCCGCAG GGATATTGGC GCCAGAGTAC CCCCGTGACA  1576
TT01       CGTGCAACAA CTAGTGCGTT TGCTTGAACG TAACCACATC ACCCCCGCAG GGGTGTTGGC GCCGGAGTAC CCCCGTGACA  1597
Consensus  CGNGCANCAN CTNGTNCGTT TGNTNGAACG NAACCACATC ACCCCCGCAG GGNTNTTGGC GCCNGAGTAC CCCCGTGACA

M1021      ACATGCGTGA TATCAAGGGC AATAACCTGA ACAAGGTGCT ATGGGAACAG GCGTTTGCCG CTTCGGTCTG GCAGAGCACC  1656
TT01       ACATGCGCGA TATCAAAGGT AATAACTTGA ATAAGGTGCT GTGGGAACAG GCGTTCGCCG CTTCGGTTTG GCGAAGCAGA  1677
Consensus  ACATGCGNGA TATCAANGGN AATAACNTGA ANAAGGTGCT NTGGGAACAG GCGTTNGCCG CTTCGGTNTG GCNNAGCANN

M1021      GATAATGACC GGCTGCTGTT CAATCTGGCG ACGAAACTGG TGGAGAACCA GGACATGGAC AAGGTCCTGC AAAGTGGCTA  1736
TT01       GATAATGACC CGTTGTTGTT CAGGCTGGCG ACGAGACTGG TGAAAAACCC GGCTGTGGTC AAGGTTCTGC AAAATGGCTA  1757
Consensus  GATAATGACC NGNTGNTGTT CANNCTGGCG ACGANACTGG TGNANAACCN GGNNNTGGNC AAGGTNCTGC AAANTGGCTA
```

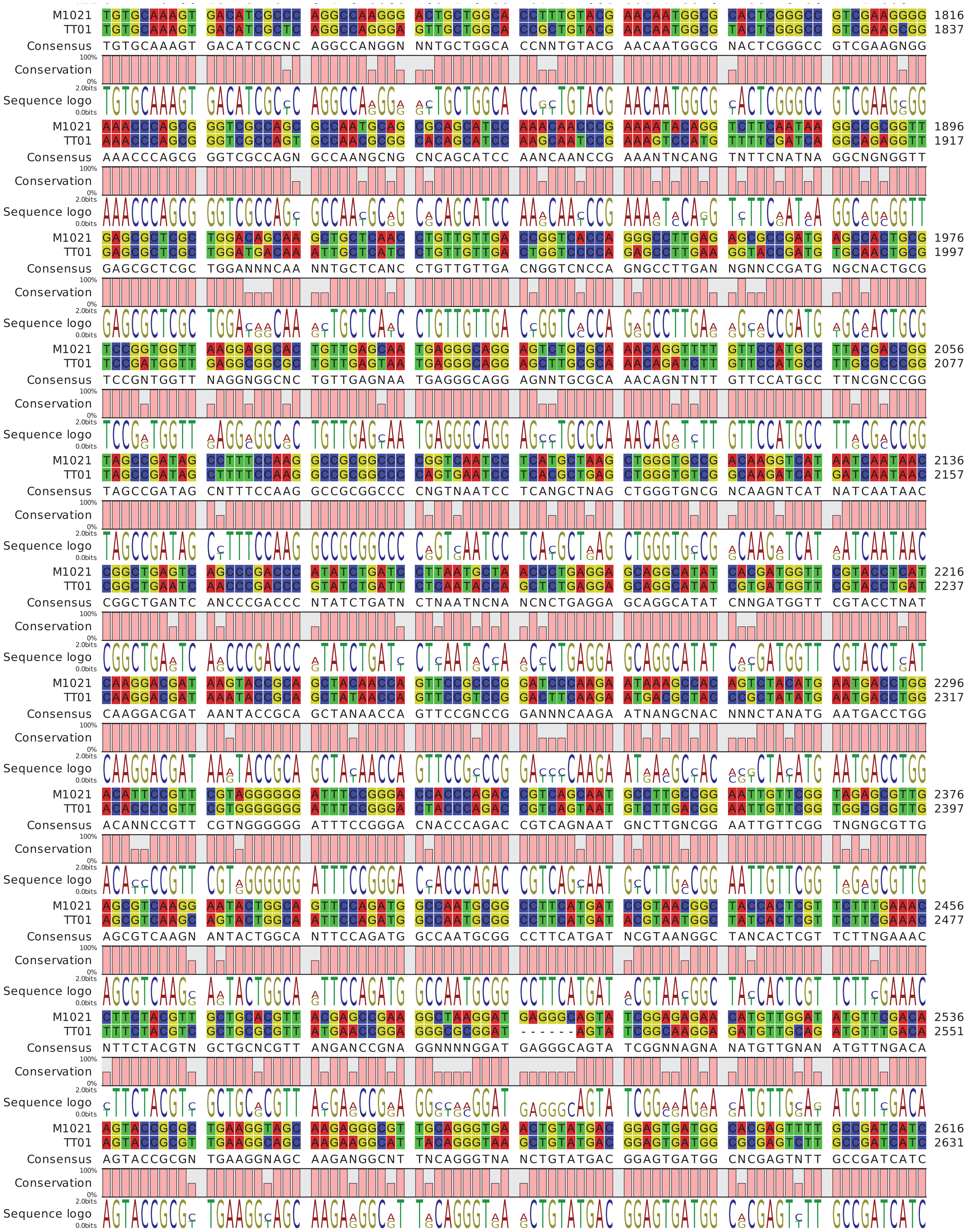
M1021 TGTGCAAAGT GACATCGCCC AGGCCAAGGG ACTGCTGGCA CCTTTGTACG AACAATGGCG CACTCGGGCC GTCGAAGGGG 1816
TT01 TGTGCAAAGT GACATCGCTC AGGCCAGGGA GTTGCTGGCA CCGCTGTACG AACAATGGCG TACTCGGGCC GTCGAAGCGG 1837
Consensus TGTGCAAAGT GACATCGCNC AGGCCANGGN NNTGCTGGCA CCNNTGTACG AACAATGGCG NACTCGGGCC GTCGAAGNGG
Conservation
Sequence logo
M1021 AAACCCAGCG GGTCGCCAGC GCCAATGCAG CGCAGCATCC AAACAACCCG AAAATACAGG TCTTCAATAA GGCCGCGGTT 1896
TT01 AAACCCAGCG GGTCGCCAGT GCCAACGCGG CACAGCATCC AAGCAATCCG AAAGTCCATG TTTTCGATCA GGCAGAGGTT 1917
Consensus AAACCCAGCG GGTCGCCAGN GCCAANGCNG CNCAGCATCC AANCAANCCG AAANTNCANG TNTTCNATNA GGCNGNGGTT
Conservation
Sequence logo
M1021 GAGCGCTCGC TGGACAGCAA GCTGCTCAAC CTGTTGTTGA CCGGTCACCA GGGCCTTGAG AGCGCCGATG AGCCACTGCG 1976
TT01 GAGCGCTCGC TGGATGACAA ATTGCTCATC CTGTTGTTGA CTGGTCCCCA GAGCCTTGAA GGTACCGATG TGCAACTGCG 1997
Consensus GAGCGCTCGC TGGANNNCAA NNTGCTCANC CTGTTGTTGA CNGGTCNCCA GNGCCTTGAN NGNNCCGATG NGCNACTGCG
Conservation
Sequence logo
M1021 TCCGGTGGTT AAGGAGGCAC TGTTGAGCAA TGAGGGCAGG AGTCTGCGCA AACAGGTTTT GTTCCATGCC TTACGACCGG 2056
TT01 TCCGATGGTT GAGGCGGCGC TGTTGAGTAA TGAGGGCAGG AGCTTGCGCA AACAGATCTT GTTCCATGCC TTGCGCCCGG 2077
Consensus TCCGNTGGTT NAGGNGGCNC TGTTGAGNAA TGAGGGCAGG AGNNTGCGCA AACAGNTNTT GTTCCATGCC TTNCGNCCGG
Conservation
Sequence logo
M1021 TAGCCGATAG CCTTTCCAAG GCCGCGGCCC CGGTCAATCC TCATGCTAAG CTGGGTGCCG ACAAGGTCAT AATCAATAAC 2136
TT01 TAGCCGATAG CTTTTCCAAG GCCGCGGCCC CAGTGAATCC TCACGCTGAG CTGGGTGTCG GCAAGATCAT GATCAATAAC 2157
Consensus TAGCCGATAG CNTTTCCAAG GCCGCGGCCC CNGTNAATCC TCANGCTNAG CTGGGTGNCG NCAAGNTCAT NATCAATAAC
Conservation
Sequence logo
M1021 CGGCTGAGTC AGCCCGACCC ATATCTGATC CTTAATGCTA ACCCTGAGGA GCAGGCATAT CACGATGGTT CGTACCTCAT 2216
TT01 CGGCTGAATC AACCCGACCC GTATCTGATT CTCAATACCA GCTCTGAGGA GCAGGCATAT CGTGATGGTT CGTACCTGAT 2237
Consensus CGGCTGANTC ANCCCGACCC NTATCTGATN CTNAATNCNA NCNCTGAGGA GCAGGCATAT CNNGATGGTT CGTACCTNAT
Conservation
Sequence logo
M1021 CAAGGACGAT AAGTACCGCA GCTACAACCA GTTCCGCCCG GATCCCAAGA ATAAAGCCAC AGTCTACATG AATGACCTGG 2296
TT01 CAAGGACGAT AAATACCGCA GCTATAACCA GTTCCGTCCG GACTTCAAGA ATGACGCTAC CCGCTATATG AATGACCTGG 2317
Consensus CAAGGACGAT AANTACCGCA GCTANAACCA GTTCCGNCCG GANNNCAAGA ATNANGCNAC NNNCTANATG AATGACCTGG
Conservation
Sequence logo
M1021 ACATTCCGTT CGTAGGGGGG ATTTCCGGGA CCACCCAGAC CGTCAGCAAT GCCTTGCCGG AATTGTTCGG TAGAGCGTTG 2376
TT01 ACACCCCGTT CGTGGGGGGG ATTTCCGGGA CTACCCAGAC CGTCAGTAAT GTCTTGACGG AATTGTTCGG TGGCGCGTTG 2397
Consensus ACANNCCGTT CGTNGGGGGG ATTTCCGGGA CNACCCAGAC CGTCAGNAAT GNCTTGNCGG AATTGTTCGG TNGNGCGTTG
Conservation
Sequence logo
M1021 AGCGTCAAGG AATACTGGCA GTTCCAGATG GCCAATGCGG CCTTCATGAT CCGTAACGGC TACCACTCGT TCTTTGAAAC 2456
TT01 AGCGTCAAGC AGTACTGGCA ATTCCAGATG GCCAATGCGG CCTTCATGAT ACGTAATGGC TATCACTCGT TCTTCGAAAC 2477
Consensus AGCGTCAAGN ANTACTGGCA NTTCCAGATG GCCAATGCGG CCTTCATGAT NCGTAANGGC TANCACTCGT TCTTNGAAAC
Conservation
Sequence logo
M1021 CTTCTACGTT GCTGCACGTT ACGAGCCGAA GGCTAAGGAT GAGGGCAGTA TCGGAGAGAA CATGTTGGAT ATGTTCGACA 2536
TT01 TTTCTACGTC GCTGCGCGTT ATGAACCGGA GGGCGCGGAT -------AGTA TCGGCAAGGA GATGTTGCAG ATGTTTGACA 2551
Consensus NTTCTACGTN GCTGCNCGTT ANGANCCGNA GGNNNNGGAT GAGGGCAGTA TCGGNNAGNA NATGTTGNAN ATGTTNGACA
Conservation
Sequence logo
M1021 AGTACCGCGC TGAAGGTAGC AAGAGGGCGT TGCAGGGTGA ACTGTATGAC GGAGTGATGG CACGAGTTTT GCCGATCATC 2616
TT01 AGTACCGCGT TGAAGGCAGC AAGAAGGCAT TACAGGGTAA GCTGTATGAC GGAGTGATGG CGCGAGTCTT GCCGATCATC 2631
Consensus AGTACCGCGN TGAAGGNAGC AAGANGGCNT TNCAGGGTNA NCTGTATGAC GGAGTGATGG CNCGAGTNTT GCCGATCATC
Conservation
Sequence logo

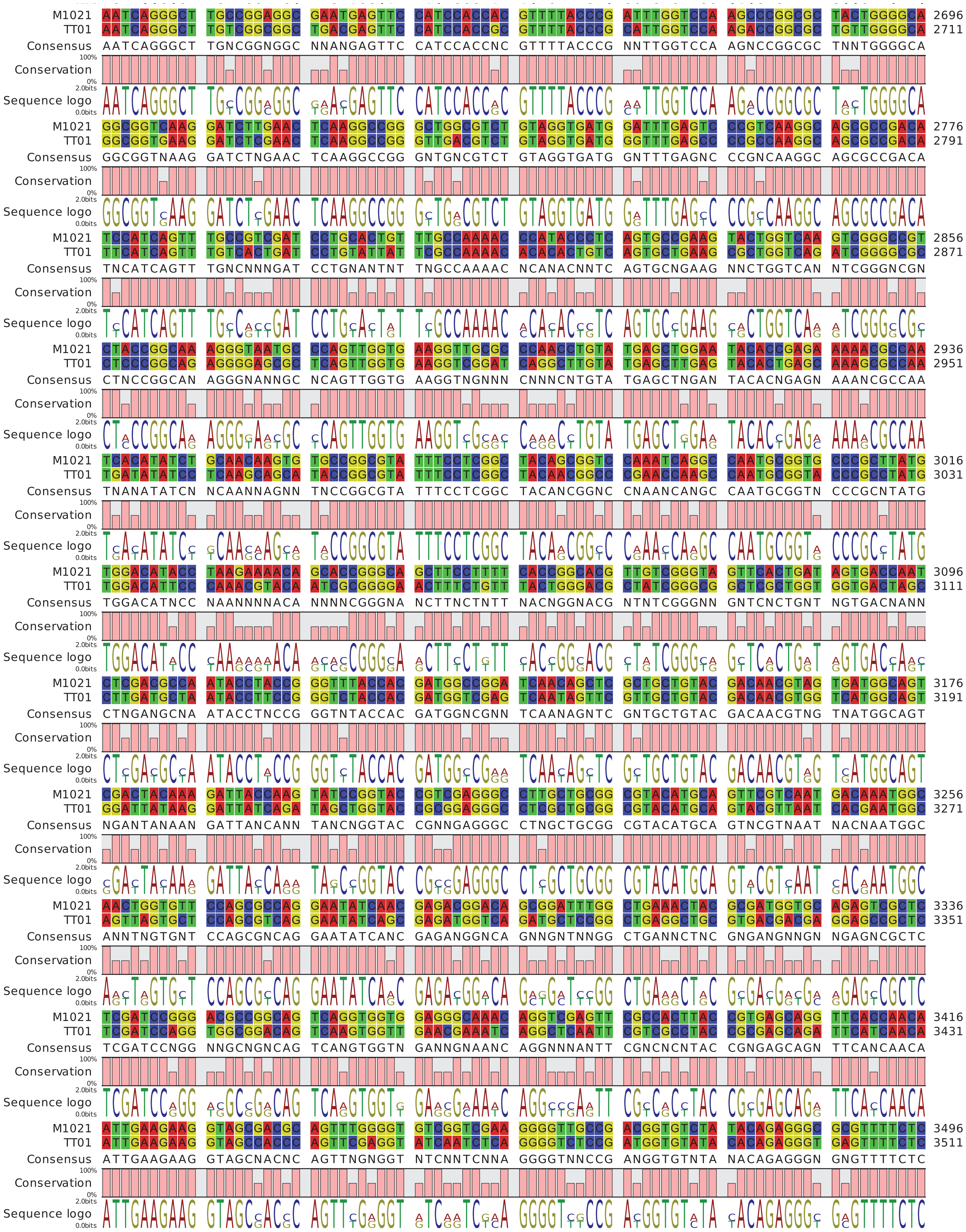
M1021 AATCAGGGCT TGCCGGAGGC GAATGAGTTC CATCCACCAC GTTTTACCCG ATTTGGTCCA AGCCCGGCGC TACTGGGGCA 2696
TT01 AATCAGGGCT TGTCGGCGGC TGACGAGTTC CATCCACCGC GTTTTACCCG CATTGGTCCA AGACCGGCGC TGTTGGGGCA 2711
Consensus AATCAGGGCT TGNCGGNGGC NNANGAGTTC CATCCACCNC GTTTTACCCG NNTTGGTCCA AGNCCGGCGC TNNTGGGGCA
M1021 GGCGGTCAAG GATCTTGAAC TCAAGGCCGG GCTGGCGTCT GTAGGTGATG GATTTGAGTC CCGTCAAGGC AGCGCCGACA 2776
TT01 GGCGGTGAAG GATCTCGAAC TCAAGGCCGG GTTGACGTCT GTAGGTGATG GGTTTGAGCC CCGCCAAGGC AGCGCCGACA 2791
Consensus GGCGGTNAAG GATCTNGAAC TCAAGGCCGG GNTGNCGTCT GTAGGTGATG GNTTTGAGNC CCGNCAAGGC AGCGCCGACA
M1021 TCCATCAGTT TGCCGTCGAT CCTGCACTGT TTGCCAAAAC CCATACCCTC AGTGCCGAAG TACTGGTCAA GTCGGGCCGT 2856
TT01 TTCATCAGTT TGTCACTGAT CCTGTATTAT TCGCCAAAAC ACACACTGTC AGTGCTGAAG CGCTGGTCAG ATCGGGGCGC 2871
Consensus TNCATCAGTT TGNCNNNGAT CCTGNANTNT TNGCCAAAAC NCANACNNTC AGTGCNGAAG NNCTGGTCAN NTCGGGNCGN
M1021 CTACCGGCAA AGGGTAATGC CCAGTTGGTG AAGGTTGCGC CCAACCTGTA TGAGCTGGAA TACACCGAGA AAAACGCCAA 2936
TT01 CTCCCGGCAG AGGGGAGCGC TCAGTTGGTG AAGGTCGGAT CAGGCTTGTA TGAGCTTGAG TACACTGAGC AAAGCGCCAA 2951
Consensus CTNCCGGCAN AGGGNANNGC NCAGTTGGTG AAGGTNGNNN CNNNCNTGTA TGAGCTNGAN TACACNGAGN AAANCGCCAA
M1021 TCACATATCT GCAACAAGTG TGCCGGCGTA TTTCCTCGGC TACAGCGGTC CAAATCAGGC CAATGCGGTG CCCGCTTATG 3016
TT01 TGATATATCC TCAAGCAGCA TACCGGCGTA TTTCCTCGGC TACAACGGCC CGAACCAAGC CAATGCGGTA CCCGCCTATG 3031
Consensus TNANATATCN NCAANNAGNN TNCCGGCGTA TTTCCTCGGC TACANCGGNC CNAANCANGC CAATGCGGTN CCCGCNTATG
M1021 TGGACATACC TAAGAAAACA GCACCGGGCA GCTTCCTTTT CACCGGCACG TTGTCGGGTA GTTCACTGAT AGTGACCAAT 3096
TT01 TGGACATTCC CAAACGTACA ATCGCGGGGA ACTTTCTGTT TACTGGGACG CTATCGGGCG GCTCGCTGGT GGTGACTAGC 3111
Consensus TGGACATNCC NAANNNNACA NNNNCGGGNA NCTTNCTNTT NACNGGNACG NTNTCGGGNN GNTCNCTGNT NGTGACNANN
M1021 CTCGACGCCA ATACCTACCG GGTTTACCAC GATGGCCGGA TCAACAGCTC GCTGCTGTAC GACAACGTAG TGATGGCAGT 3176
TT01 CTTGATGCTA ATACCTTCCG GGTCTACCAC GATGGTCGAG TCAATAGTTC GTTGCTGTAC GACAACGTGG TCATGGCAGT 3191
Consensus CTNGANGCNA ATACCTNCCG GGTNTACCAC GATGGNCGNN TCAANAGNTC GNTGCTGTAC GACAACGTNG TNATGGCAGT
M1021 CGACTACAAA GATTACCAAG TATCCGGTAC CGTCGAGGGC CTTGCTGCGG CGTACATGCA GTTCGTCAAT GACAAATGGC 3256
TT01 GGATTATAAG GATTATCAGA TAGCTGGTAC CGCGGAGGGC CTCGCTGCGG CGTACATGCA GTACGTTAAT CACGAATGGC 3271
Consensus NGANTANAAN GATTANCANN TANCNGGTAC CGNNGAGGGC CTNGCTGCGG CGTACATGCA GTNCGTNAAT NACNAATGGC
M1021 AACTGGTGTT CCAGCGCCAG GAATATCAAC GAGACGGACA GCGGATTTGG CTGAAACTAC GCGATGGTGC AGAGTCGCTC 3336
TT01 AGTTAGTGCT CCAGCGTCAG GAATATCAGC GAGATGGTCA GATGCTCCGG CTGAGGCTGC GTGACGACGA GGAGCCGCTC 3351
Consensus ANNTNGTGNT CCAGCGNCAG GAATATCANC GAGANGGNCA GNNGNTNNGG CTGANNCTNC GNGANGNNGN NGAGNCGCTC
M1021 TCGATCCGGG ACGCCGGCAG TCAGGTGGTG GAGGGCAAAC AGGTCGAGTT CGCCACTTAC CGTGAGCAGG TTCACCAACA 3416
TT01 TCGATCCAGG TGGCGGACAG TCAAGTGGTT GAACGAAATC AGGCTCAATT CGTCGCCTAC CGCGAGCAGA TTCATCAACA 3431
Consensus TCGATCCNGG NNGCNGNCAG TCANGTGGTN GANNGNAANC AGGNNNANTT CGNCNCNTAC CGNGAGCAGN TTCANCAACA
M1021 ATTGAAGAAG GTAGCGACGC AGTTTGGGGT GTCGGTCGAA GGGGTTGCCG ACGGTGTCTA TACAGAGGGC GCGTTTTCTC 3496
TT01 ATTGAAGAAG GTAGCCACCC AGTTCGAGGT ATCAATCTCA GGGGTCTCCG ATGGTGTATA CACAGAGGGT GAGTTTTCTC 3511
Consensus ATTGAAGAAG GTAGCNACNC AGTTNGNGGT NTCNNTCNNA GGGGTNNCCG ANGGTGTNTA NACAGAGGGN GNGTTTTCTC
Conservation
100%
0%
Sequence logo
2.0bits
0.0bits

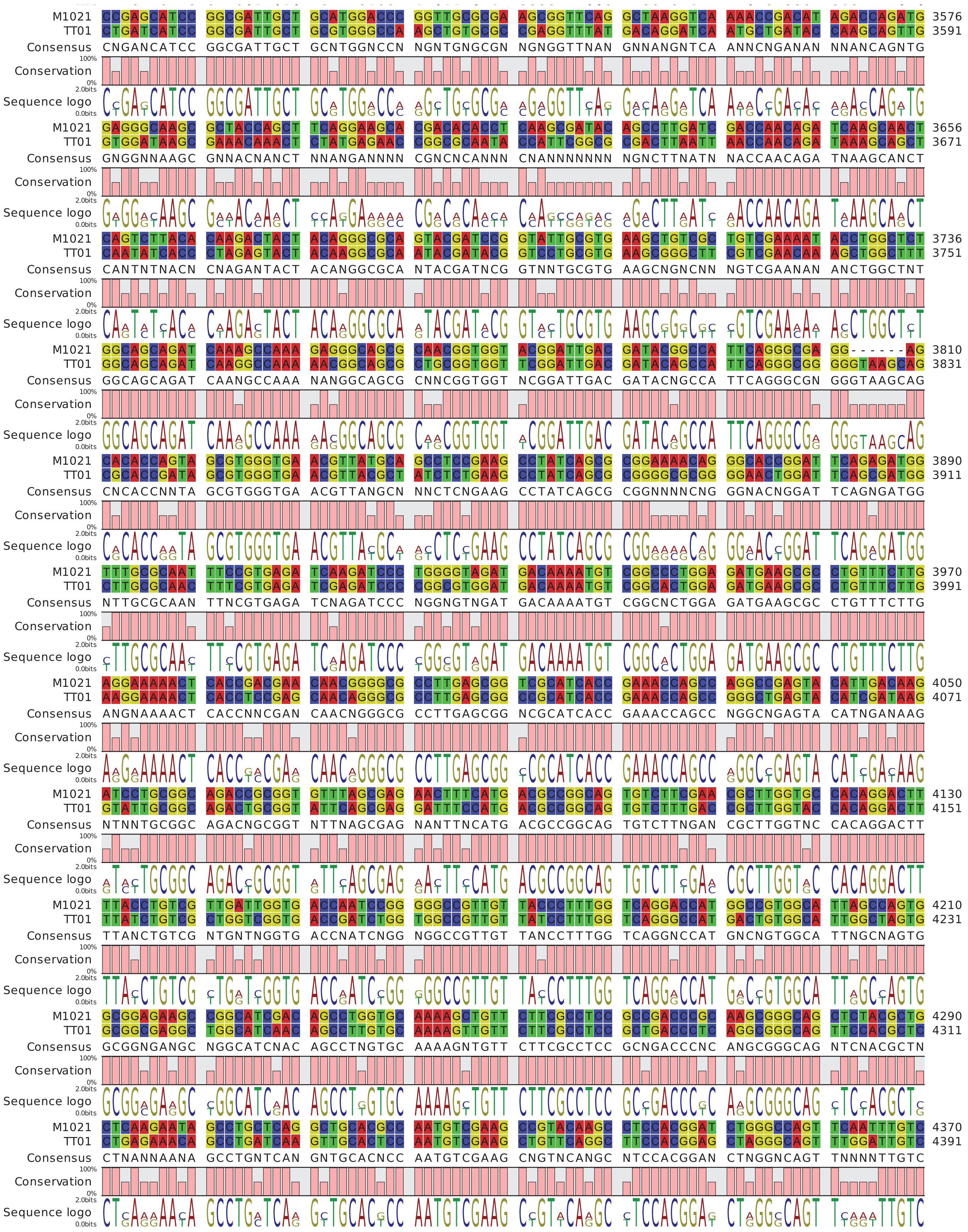
M1021 CCGAGCATCC GGCGATTGCT GCATGGACCC GGTTGCGCGA AGCGGTTCAG GCTAAGGTCA AAACCGACAT AGACCAGATG 3576
TT01 CTGATCATCC GGCGATTGCT GCGTGGGCCA AGCTGTGCGC CGAGGTTTAT GACAGGATCA ATGCTGATAC CAAGCAGTTG 3591
Consensus CNGANCATCC GGCGATTGCT GCNTGGNCCN NGNTGNGCGN NGNGGTTNAN GNNANGNTCA ANNCNGANAN NNANCAGNTG
Conservation
Sequence logo
M1021 GAGGGCAAGC GCTACCAGCT TCAGGAAGCA CGACACACCT CAAGCGATAC AGCCTTGATC GACCAACAGA TCAAGCAACT 3656
TT01 GTGGATAAGC GAAACAAACT CTATGAGAAC CGGCGCAATA CCATTCGGCG CGACTTAATT AACCAACAGA TAAAGCAGCT 3671
Consensus GNGGNNAAGC GNNACNANCT NNANGANNNN CGNCNCANNN CNANNNNNNN NGNCTTNATN NACCAACAGA TNAAGCANCT
Conservation
Sequence logo
M1021 CAGTCTTACA CAAGACTACT ACAGGGCGCA GTACGATCCG GTATTGCGTG AAGCTGTCGC TGTCGAAAAT ACCTGGCTCT 3736
TT01 CAATATCACC CTAGAGTACT ACAAGGCGCA ATACGATACG GTCCTGCGTG AAGCGGGCTT CGTCGAACAA AGCTGGCTTT 3751
Consensus CANTNTNACN CNAGANTACT ACANGGCGCA NTACGATNCG GTNNTGCGTG AAGCNGNCNN NGTCGAANAN ANCTGGCTNT
Conservation
Sequence logo
M1021 GGCAGCAGAT CAAAGCCAAA GAGGGCAGCG CAACGGTGGT ACGGATTGAC GATACGGCCA TTCAGGGCGA GG------AG 3810
TT01 GGCAGCAGAT CAAGGCCAAA AACGGCAGCG CTGCGGTGGT TCGGATTGAC GATACAGCCA TTCAGGGCGG GGGTAAGCAG 3831
Consensus GGCAGCAGAT CAANGCCAAA NANGGCAGCG CNNCGGTGGT NCGGATTGAC GATACNGCCA TTCAGGGCGN GGGTAAGCAG
Conservation
Sequence logo
M1021 CACACCAGTA GCGTGGGTGA ACGTTATGCA GCCTCCGAAG CCTATCAGCG CGGAAAACAG GGCACCGGAT TCAGAGATGG 3890
TT01 CGCACCGATA GCGTGGGTGA ACGTTACGCT ATCTCTGAAG CCTATCAGCG CGGGGCGCGG GGAACTGGAT TCAGCGATGG 3911
Consensus CNCACCNNTA GCGTGGGTGA ACGTTANGCN NNCTCNGAAG CCTATCAGCG CGGNNNNCNG GGNACNGGAT TCAGNGATGG
Conservation
Sequence logo
M1021 TTTGCGCAAT TTCCGTGAGA TCAAGATCCC TGGGGTAGAT GACAAAATGT CGGCCCTGGA GATGAAGCGC CTGTTTCTTG 3970
TT01 CTTGCGCAAC TTTCGTGAGA TCGAGATCCC CGGCGTGGAT GACAAAATGT CGGCACTGGA GATGAAGCGC CTGTTTCTTG 3991
Consensus NTTGCGCAAN TTNCGTGAGA TCNAGATCCC NGGNGTNGAT GACAAAATGT CGGCNCTGGA GATGAAGCGC CTGTTTCTTG
Conservation
Sequence logo
M1021 AGGAAAAACT CACCGACGAA CAACGGGGCG CCTTGAGCGG TCGCATCACC GAAACCAGCC AGGCCGAGTA CATTGACAAG 4050
TT01 AAGGAAAACT CACCTCCGAG CAACAGGGCG CCTTGAGCGG CCGCATCACC GAAACCAGCC GGGCTGAGTA CATCGATAAG 4071
Consensus ANGNAAAACT CACCNNCGAN CAACNGGGCG CCTTGAGCGG NCGCATCACC GAAACCAGCC NGGCNGAGTA CATNGANAAG
Conservation
Sequence logo
M1021 ATCCTGCGGC AGACCGCGGT GTTTAGCGAG AACTTTCATG ACGCCGGCAG TGTCTTCGAA CGCTTGGTGC CACAGGACTT 4130
TT01 GTATTGCGGC AGACTGCGGT ATTCAGCGAG GATTTCCATG ACGCCGGCAG TGTCTTTGAC CGCTTGGTAC CACAGGACTT 4151
Consensus NTNNTGCGGC AGACNGCGGT NTTNAGCGAG NANTTNCATG ACGCCGGCAG TGTCTTNGAN CGCTTGGTNC CACAGGACTT
Conservation
Sequence logo
M1021 TTACCTGTCG TTGATTGGTG ACCAATCCGG GGGCCGTTGT TACCCTTTGG TCAGGACCAT GGCCGTGGCA TTAGCCAGTG 4210
TT01 TTATCTGTCG CTGGTCGGTG ACCGATCTGG TGGCCGTTGT TATCCTTTGG TCAGGGCCAT GACTGTGGCA TTGGCTAGTG 4231
Consensus TTANCTGTCG NTGNTNGGTG ACCNATCNGG NGGCCGTTGT TANCCTTTGG TCAGGNCCAT GNCNGTGGCA TTNGCNAGTG
Conservation
Sequence logo
M1021 GCGGAGAAGC CGGCATCGAC AGCCTGGTGC AAAAGCTGTT CTTCGCCTCC GCCGACCCGC AAGCGGGCAG CTCTACGCTG 4290
TT01 GCGGCGAGGC TGGCATCAAC AGCCTTGTGC AAAAGTTGTT CTTCGCCTCC GCTGACCCTC AGGCGGGCAG TTCCACGCTC 4311
Consensus GCGGNGANGC NGGCATCNAC AGCCTNGTGC AAAAGNTGTT CTTCGCCTCC GCNGACCCNC ANGCGGGCAG NTCNACGCTN
Conservation
Sequence logo
M1021 CTCAAGAATA GCCTGCTCAG GCTGCACGCC AATGTCGAAG CCGTACAAGC CTCCACGGAT CTGGGCCAGT TCAATTTGTC 4370
TT01 CTGAGAAACA GCCTGATCAA GTTGCACTCC AATGTCGAAG CTGTTCAGGC TTCCACGGAG CTAGGGCAGT TTGGATTGTC 4391
Consensus CTNANNAANA GCCTGNTCAN GNTGCACNCC AATGTCGAAG CNGTNCANGC NTCCACGGAN CTNGGNCAGT TNNNNTTGTC
Conservation
Sequence logo

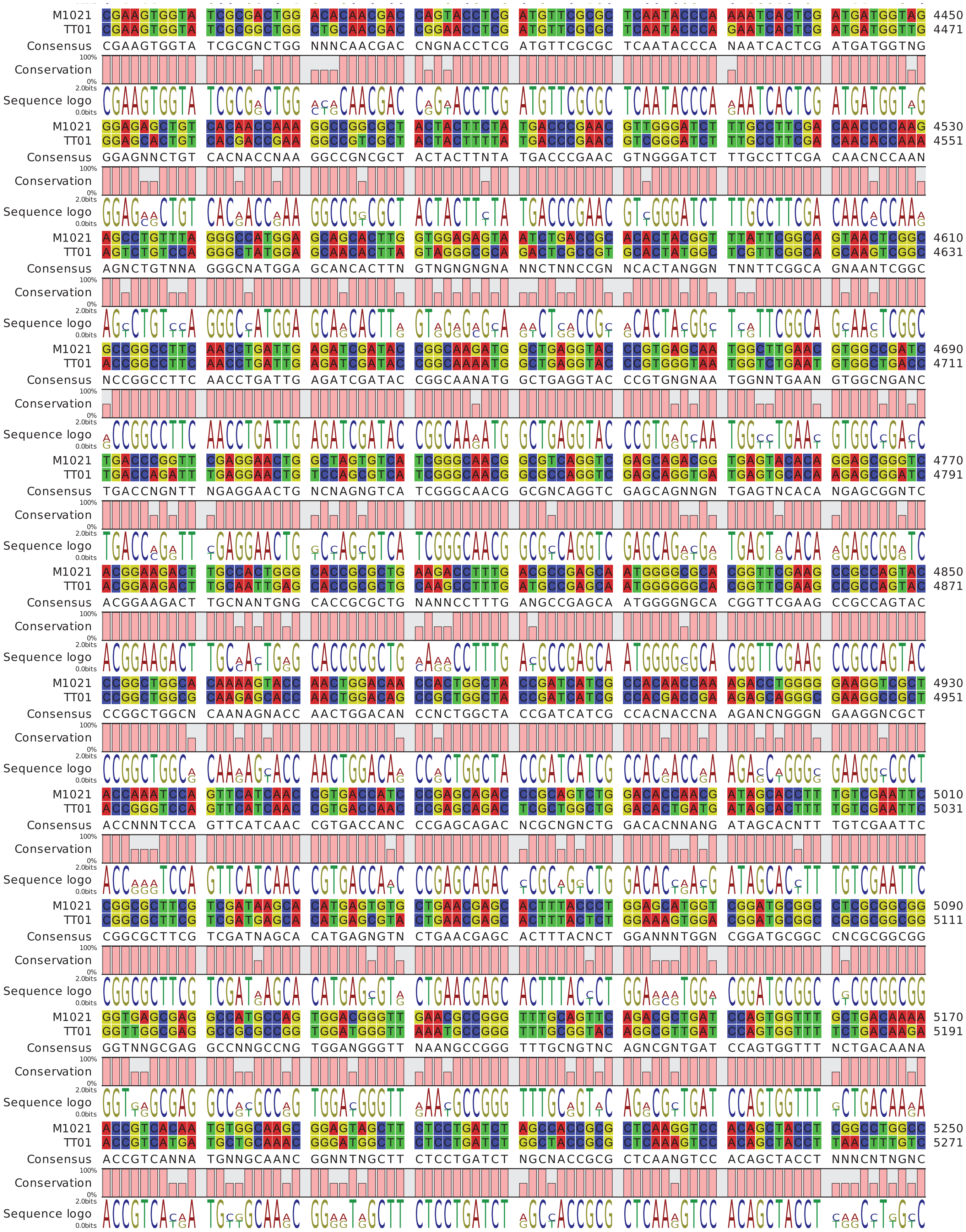
M1021 CGAAGTGGTA TCGCGACTGG ACACAACGAC CAGTACCTCG ATGTTCGCGC TCAATACCCA AAATCACTCG ATGATGGTAG 4450
TT01 CGAAGTGGTA TCGCGGCTGG CTGCAACGAC CGGAACCTCG ATGTTCGCGC TCAATACCCA GAATCACTCG ATGATGGTTG 4471
Consensus CGAAGTGGTA TCGCGNCTGG NNNCAACGAC CNGNACCTCG ATGTTCGCGC TCAATACCCA NAATCACTCG ATGATGGTNG
Conservation
Sequence logo
M1021 GGAGAGCTGT CACAACCAAA GGCCGGCGCT ACTACTTCTA TGACCCGAAC GTTGGGATCT TTGCCTTCGA CAACCCCAAG 4530
TT01 GGAGCACTGT CACGACCGAA GGCCGTCGCT ACTACTTTTA TGACCCGAAC GTCGGGATCT TTGCCTTCGA CAACACCAAA 4551
Consensus GGAGNNCTGT CACNACCNAA GGCCGNCGCT ACTACTTNTA TGACCCGAAC GTNGGGATCT TTGCCTTCGA CAACNCCAAN
M1021 AGCCTGTTTA GGGCCATGGA GCAGCACTTG GTGGAGAGTA ATCTGACCGC ACACTACGGT TTATTCGGCA GTAACTCGGC 4610
TT01 AGTCTGTCCA GGGCTATGGA GCAACACTTA GTAGGGCGCA GACTCGCCGT GCACTATGGC TCGTTCGGCA GCAAGTCGGC 4631
Consensus AGNCTGTNNA GGGCNATGGA GCANCACTTN GTNGNGNGNA NNCTNNCCGN NCACTANGGN TNNTTCGGCA GNAANTCGGC
M1021 GCCGGCCTTC AACCTGATTG AGATCGATAC CGGCAAGATG GCTGAGGTAC CCGTGAGCAA TGGCTTGAAC GTGGCCGATC 4690
TT01 ACCGGCCTTC AACCTGATTG AGATCGATAC CGGCAAAATG GCTGAGGTAC CCGTGGGTAA TGGTCTGAAT GTGGCTGACC 4711
Consensus NCCGGCCTTC AACCTGATTG AGATCGATAC CGGCAANATG GCTGAGGTAC CCGTGNGNAA TGGNNTGAAN GTGGCNGANC
M1021 TGACCCGGTT CGAGGAACTG GCTAGTGTCA TCGGGCAACG GCGTCAGGTC GAGCAGACGG TGAGTACACA GGAGCGGGTC 4770
TT01 TGACCAGATT TGAGGAACTG TCCAGCGTCA TCGGGCAACG GCGCCAGGTC GAGCAGGTGA TGAGTGCACA AGAGCGGATC 4791
Consensus TGACCNGNTT NGAGGAACTG NCNAGNGTCA TCGGGCAACG GCGNCAGGTC GAGCAGNNGN TGAGTNCACA NGAGCGGNTC
M1021 ACGGAAGACT TGCCACTGGG CACCGCGCTG AAGACCTTTG ACGCCGAGCA ATGGGGCGCA CGGTTCGAAG CCGCCAGTAC 4850
TT01 ACGGAAGACT TGCAATTGAG CACCGCGCTG CAAGCCTTTG ATGCCGAGCA ATGGGGGGCA CGGTTCGAAG CCGCCAGTAC 4871
Consensus ACGGAAGACT TGCNANTGNG CACCGCGCTG NANNCCTTTG ANGCCGAGCA ATGGGGNGCA CGGTTCGAAG CCGCCAGTAC
M1021 CCGGCTGGCA CAAAAGTACC AACTGGACAA CCACTGGCTA CCGATCATCG CCACAACCAA AGACCTGGGG GAAGGTCGCT 4930
TT01 CCGGCTGGCG CAAGAGCACC AACTGGACAG CCGCTGGCTA CCGATCATCG CCACGACCGA AGAGCAGGGC GAAGGCCGCT 4951
Consensus CCGGCTGGCN CAANAGNACC AACTGGACAN CCNCTGGCTA CCGATCATCG CCACNACCNA AGANCNGGGN GAAGGNCGCT
M1021 ACCAAATCCA GTTCATCAAC CGTGACCATC CCGAGCAGAC CCGCAGTCTG GACACCAAGG ATAGCACCTT TGTCGAATTC 5010
TT01 ACCGGGTCCA GTTCATCAAC CGTGACCAAC CCGAGCAGAC TCGCTGGCTG GACACTGATG ATAGCACTTT TGTCGAATTC 5031
Consensus ACCNNNTCCA GTTCATCAAC CGTGACCANC CCGAGCAGAC NCGCNGNCTG GACACNNANG ATAGCACNTT TGTCGAATTC
M1021 CGGCGCTTCG TCGATAAGCA CATGAGTGTG CTGAACGAGC ACTTTACCCT GGAGCATGGT CGGATGCGGC CTCGCGGCGG 5090
TT01 CGGCGCTTCG TCGATGAGCA CATGAGCGTA CTGAACGAGC ACTTTACTCT GGAAAGTGGA CGGATGCGGC CGCGCGGCGG 5111
Consensus CGGCGCTTCG TCGATNAGCA CATGAGNGTN CTGAACGAGC ACTTTACNCT GGANNNTGGN CGGATGCGGC CNCGCGGCGG
M1021 GGTGAGCGAG GCCATGCCAG TGGACGGGTT GAACGCCGGG TTTGCAGTTC AGACGCTGAT CCAGTGGTTT GCTGACAAAA 5170
TT01 GGTTGGCGAG GCCGCGCCGG TGGATGGGTT AAATGCCGGG TTTGCGGTAC AGGCGTTGAT CCAGTGGTTT TCTGACAAGA 5191
Consensus GGTNNGCGAG GCCNNGCCNG TGGANGGGTT NAANGCCGGG TTTGCNGTNC AGNCGNTGAT CCAGTGGTTT NCTGACAANA
M1021 ACCGTCACAA TGTGGCAAGC GGAGTAGCTT CTCCTGATCT AGCCACCGCG CTCAAGGTCC ACAGCTACCT CGGCCTGGCC 5250
TT01 ACCGTCATGA TGCTGCAAAC GGGATGGCTT CTCCTGATCT GGCTACCGCG CTCAAAGTCC ACAGCTACCT TAACTTTGTC 5271
Consensus ACCGTCANNA TGNNGCAANC GGNNTNGCTT CTCCTGATCT NGCNACCGCG CTCAANGTCC ACAGCTACCT NNNCNTNGNC

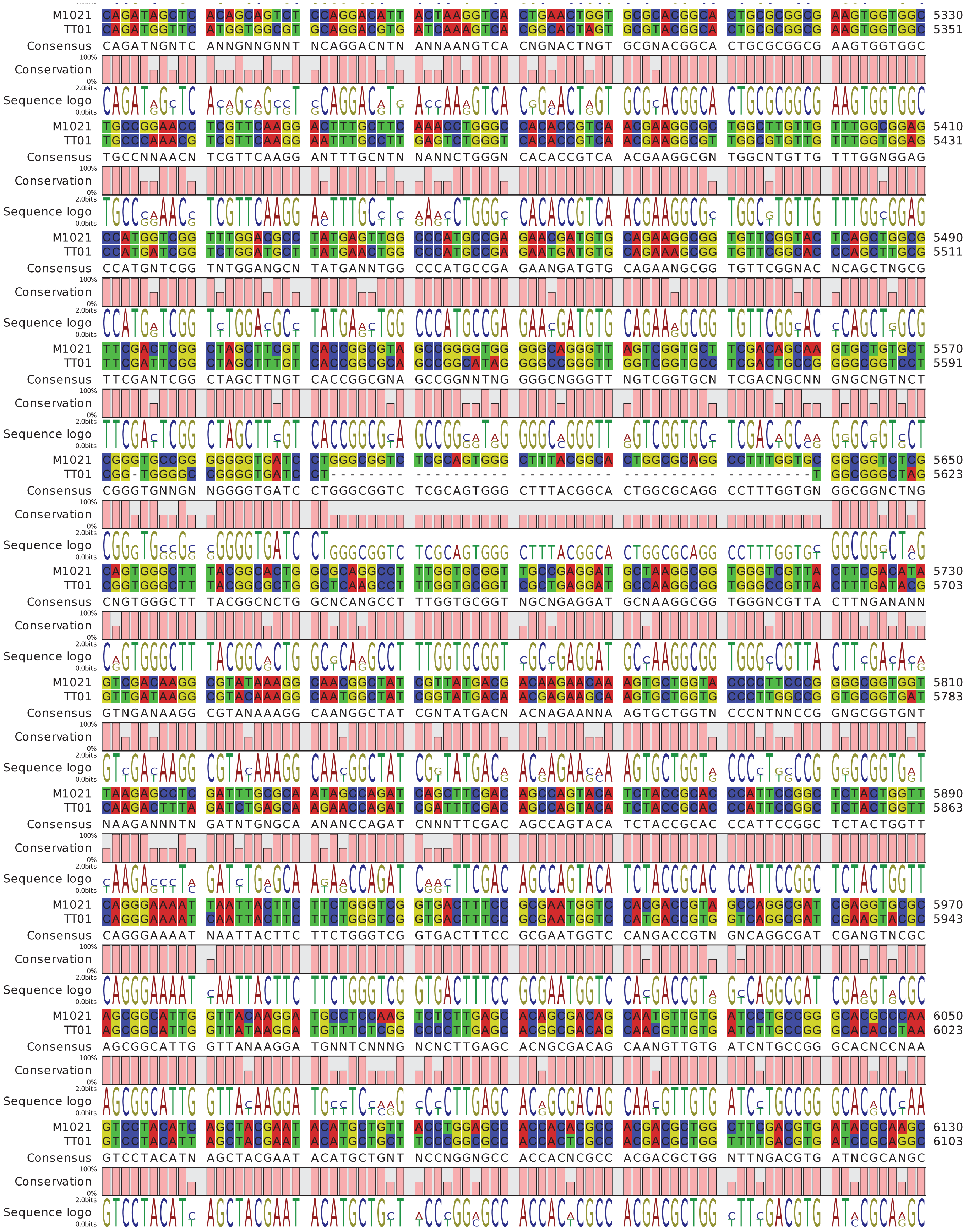
M1021 CAGATAGCTC ACAGCAGTCT CCAGGACATT ACTAAGGTCA CTGAACTGGT GCGCACGGCA CTGCGCGGCG AAGTGGTGGC 5330
TT01 CAGATGGTTC ATGGTGGCGT GCAGGACGTG ATCAAAGTCA CGGCACTAGT GCGTACGGCA CTGCGCGGCG AAGTGGTGGC 5351
Consensus CAGATNGNTC ANNGNNGNNT NCAGGACNTN ANNAANGTCA CNGNACTNGT GCGNACGGCA CTGCGCGGCG AAGTGGTGGC
Conservation
Sequence logo
M1021 TGCCGGAACC TCGTTCAAGG ACTTTGCTTC AAACCTGGGC CACACCGTCA ACGAAGGCGC TGGCTTGTTG TTTGGCGGAG 5410
TT01 TGCCCAAACG TCGTTCAAGG AATTTGCCTT GAGTCTGGGT CACACCGTCA ACGAAGGCGT TGGCGTGTTG TTTGGTGGAG 5431
Consensus TGCCNNAACN TCGTTCAAGG ANTTTGCNTN NANNCTGGGN CACACCGTCA ACGAAGGCGN TGGCNTGTTG TTTGGNGGAG
Conservation
Sequence logo
M1021 CCATGGTCGG TTTGGACGCC TATGAGTTGG CCCATGCCGA GAACGATGTG CAGAAGGCGG TGTTCGGTAC TCAGCTGGCG 5490
TT01 CCATGATCGG TCTGGATGCT TATGAACTGG CCCATGCCGA GAATGATGTG CAGAAAGCGG TGTTCGGCAC CCAGCTTGCG 5511
Consensus CCATGNTCGG TNTGGANGCN TATGANNTGG CCCATGCCGA GAANGATGTG CAGAANGCGG TGTTCGGNAC NCAGCTNGCG
Conservation
Sequence logo
M1021 TTCGACTCGG CTAGCTTCGT CACCGGCGTA GCCGGGGTGG GGGCAGGGTT AGTCGGTGCT TCGACAGCAA GTGCTGTGCT 5570
TT01 TTCGATTCGG CTAGCTTTGT CACCGGCGCA GCCGGCATAG GGGCGGGGTT GGTCGGTGCC TCGACTGCCG GGGCGGTCCT 5591
Consensus TTCGANTCGG CTAGCTTNGT CACCGGCGNA GCCGGNNTNG GGGCNGGGTT NGTCGGTGCN TCGACNGCNN GNGCNGTNCT
Conservation
Sequence logo
M1021 CGGGTGCCGG GGGGGTGATC CTGGGCGGTC TCGCAGTGGG CTTTACGGCA CTGGCGCAGG CCTTTGGTGC GGCGGTCTCG 5650
TT01 CGG-TGGGGC CGGGGTGATC CT-------- ---------- ---------- ---------- ---------T GGCGGGCTAG 5623
Consensus CGGGTGNNGN NGGGGTGATC CTGGGCGGTC TCGCAGTGGG CTTTACGGCA CTGGCGCAGG CCTTTGGTGN GGCGGNCTNG
Conservation
Sequence logo
M1021 CAGTGGGCTT TACGGCACTG GCGCAGGCCT TTGGTGCGGT TGCCGAGGAT GCTAAGGCGG TGGGTCGTTA CTTCGACATA 5730
TT01 CGGTGGGCTT TACGGCGCTG GCTCAAGCCT TTGGTGCGGT CGCTGAGGAT GCCAAGGCGG TGGGCCGTTA CTTTGATACG 5703
Consensus CNGTGGGCTT TACGGCNCTG GCNCANGCCT TTGGTGCGGT NGCNGAGGAT GCNAAGGCGG TGGGNCGTTA CTTNGANANN
Conservation
Sequence logo
M1021 GTCGACAAGG CGTATAAAGG CAACGGCTAT CGTTATGACG ACAAGAACAA AGTGCTGGTA CCCGTTCCCG GGGCGGTGGT 5810
TT01 GTTGATAAGG CGTACAAAGG CAATGGCTAT CGGTATGACA ACGAGAAGCA AGTGCTGGTG CCCTTGGCCG GTGCGGTGAT 5783
Consensus GTNGANAAGG CGTANAAAGG CAANGGCTAT CGNTATGACN ACNAGAANNA AGTGCTGGTN CCCNTNNCCG GNGCGGTGNT
Conservation
Sequence logo
M1021 TAAGAGCCTC GATTTGCGCA ATAGCCAGAT CAGCTTCGAC AGCCAGTACA TCTACCGCAC CCATTCCGGC TCTACTGGTT 5890
TT01 CAAGACTTTA GATCTGAGCA AGAACCAGAT CGATTTCGAC AGCCAGTACA TCTACCGCAC CCATTCCGGC TCTACTGGTT 5863
Consensus NAAGANNNTN GATNTGNGCA ANANCCAGAT CNNNTTCGAC AGCCAGTACA TCTACCGCAC CCATTCCGGC TCTACTGGTT
Conservation
Sequence logo
M1021 CAGGGAAAAT TAATTACTTC TTCTGGGTCG GTGACTTTCC GCGAATGGTC CACGACCGTA GCCAGGCGAT CGAGGTGCGC 5970
TT01 CAGGGAAAAT CAATTACTTC TTCTGGGTCG GTGACTTTCC GCGAATGGTC CATGACCGTG GTCAGGCGAT CGAAGTACGC 5943
Consensus CAGGGAAAAT NAATTACTTC TTCTGGGTCG GTGACTTTCC GCGAATGGTC CANGACCGTN GNCAGGCGAT CGANGTNCGC
Conservation
Sequence logo
M1021 AGCGGCATTG GTTACAAGGA TGCCTCCAAG TCTCTTGAGC ACAGCGACAG CAATGTTGTG ATCGTGCCGG GCACGCCCAA 6050
TT01 AGCGGCATTG GTTATAAGGA TGTTTCTCGG CCCCTTGAGC ACGGCGACAG CAACGTTGTG ATCTTGCCGG GCACACCTAA 6023
Consensus AGCGGCATTG GTTANAAGGA TGNNTCNNNG NCNCTTGAGC ACNGCGACAG CAANGTTGTG ATCNTGCCGG GCACNCCNAA
Conservation
Sequence logo
M1021 GTCCTACATC AGCTACGAAT ACATGCTGTT ACCTGGAGCC ACCACACGCC ACGACGCTGG CTTCGACGTG ATACGCAAGC 6130
TT01 GTCCTACATT AGCTACGAAT ACATGCTGCT TCCGGGCGCC ACCACTCGCC ACGACGCTGG TTTTGACGTG ATCCGCAGGC 6103
Consensus GTCCTACATN AGCTACGAAT ACATGCTGNT NCCNGGNGCC ACCACNCGCC ACGACGCTGG NTTNGACGTG ATNCGCANGC
Conservation
Sequence logo

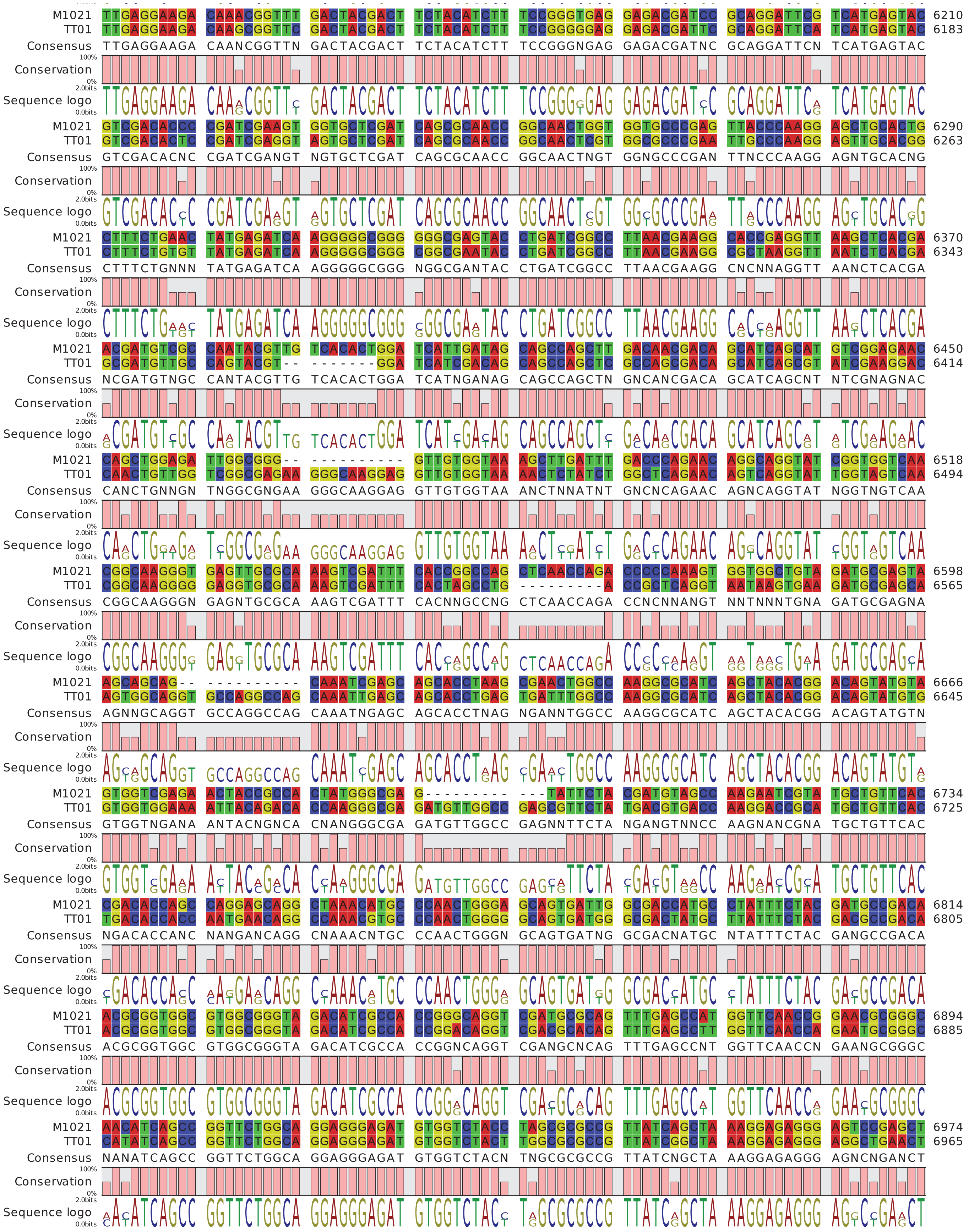
M1021 TTGAGGAAGA CAAACGGTTT GACTACGACT TCTACATCTT TCCGGGTGAG GAGACGATCC GCAGGATTCG TCATGAGTAC 6210
TT01 TTGAGGAAGA CAAGCGGTTC GACTACGACT TCTACATCTT TCCGGGGGAG GAGACGATTC GCAGGATTCA TCATGAGTAC 6183
Consensus TTGAGGAAGA CAANCGGTTN GACTACGACT TCTACATCTT TCCGGGNGAG GAGACGATNC GCAGGATTCN TCATGAGTAC
M1021 GTCGACACCC CGATCGAAGT GGTGCTCGAT CAGCGCAACC GGCAACTGGT GGTGCCCGAG TTACCCAAGG AGCTGCACTG 6290
TT01 GTCGACACTC CGATCGAGGT AGTGCTCGAT CAGCGCAACC GGCAACTCGT GGCGCCCGAA TTGCCCAAGG AGTTGCACGG 6263
Consensus GTCGACACNC CGATCGANGT NGTGCTCGAT CAGCGCAACC GGCAACTNGT GGNGCCCGAN TTNCCCAAGG AGNTGCACNG
M1021 CTTTCTGAAC TATGAGATCA AGGGGGCGGG GGGCGAGTAC CTGATCGGCC TTAACGAAGG CACCGAGGTT AAGCTCACGA 6370
TT01 CTTTCTGTGT TATGAGATCA AGGGGGCGGG CGGCGAATAC CTGATCGGCC TTAACGAAGG CGCTAAGGTT AATCTCACGA 6343
Consensus CTTTCTGNNN TATGAGATCA AGGGGGCGGG NGGCGANTAC CTGATCGGCC TTAACGAAGG CNCNNAGGTT AANCTCACGA
M1021 ACGATGTCGC CAATACGTTG TCACACTGGA TCATTGATAG CAGCCAGCTT GACAACGACA GCATCAGCAT GTCGGAGAAC 6450
TT01 GCGATGTTGC CAGTACGT-- -------GGA TCATCGACAG CAGCCAGCTC GCCAGCGACA GCATCAGCGT ATCGAAGGAC 6414
Consensus NCGATGTNGC CANTACGTTG TCACACTGGA TCATNGANAG CAGCCAGCTN GNCANCGACA GCATCAGCNT NTCGNAGNAC
M1021 CAGCTGGAGA TTGGCGGG-- ---------- GTTGTGGTAA AGCTTGATTT GACCCAGAAC AGGCAGGTAT CGGTGGTCAA 6518
TT01 CAACTGTTGG TCGGCGAGAA GGGCAAGGAG GTTGTGGTAA AACTCTATCT GGCTCAGAAC AGTCAGGTAT TGGTAGTCAA 6494
Consensus CANCTGNNGN TNGGCGNGAA GGGCAAGGAG GTTGTGGTAA ANCTNNATNT GNCNCAGAAC AGNCAGGTAT NGGTNGTCAA
M1021 CGGCAAGGGT GAGTTGCGCA AAGTCGATTT CACCGGCCAG CTCAACCAGA CCCCCAAAGT GGTGGCTGTA GATGCGAGTA 6598
TT01 CGGCAAGGGG GAGGTGCGCA AAGTCGATTT CACTAGCCTG ---------A CCGCTCAGGT AATAAGTGAA GATGCGAGCA 6565
Consensus CGGCAAGGGN GAGNTGCGCA AAGTCGATTT CACNNGCCNG CTCAACCAGA CCNCNNANGT NNTNNNTGNA GATGCGAGNA
M1021 AGCAGCAG-- ---------- CAAATCGAGC AGCACCTAAG CGAACTGGCC AAGGCGCATC AGCTACACGG ACAGTATGTA 6666
TT01 AGTGGCAGGT GCCAGGCCAG CAAATTGAGC AGCACCTGAG TGATTTGGCC AAGGCGCATC AGCTACACGG ACAGTATGTG 6645
Consensus AGNNGCAGGT GCCAGGCCAG CAAATNGAGC AGCACCTNAG NGANNTGGCC AAGGCGCATC AGCTACACGG ACAGTATGTN
M1021 GTGGTCGAGA ACTACCGCCA CTATGGGCGA G--------- ---TATTCTA CGATGTAGCC AAGAATCGTA TGCTGTTCAC 6734
TT01 GTGGTGGAAA ATTACAGACA CCAAGGGCGA GATGTTGGCC GAGCGTTCTA TGACGTGACC AAGGACCGCA TGCTGTTCAC 6725
Consensus GTGGTNGANA ANTACNGNCA CNANGGGCGA GATGTTGGCC GAGNNTTCTA NGANGTNNCC AAGNANCGNA TGCTGTTCAC
M1021 CGACACCAGC CAGGAGCAGG CTAAACATGC CCAACTGGGA GCAGTGATTG GCGACCATGC GTATTTCTAC GATGCCGACA 6814
TT01 TGACACCACC AATGAACAGG CCAAACGTGC CCAACTGGGG GCAGTGATGG GCGACTATGC TTATTTCTAC GACGCCGACA 6805
Consensus NGACACCANC NANGANCAGG CNAAACNTGC CCAACTGGGN GCAGTGATNG GCGACNATGC NTATTTCTAC GANGCCGACA
M1021 ACGCGGTGGC GTGGCGGGTA GACATCGCCA CCGGGCAGGT CGATGCGCAG TTTGAGCCAT GGTTCAACCG GAACGCGGGC 6894
TT01 ACGCGGTGGC GTGGCGGGTA GACATCGCCA CCGGACAGGT CGACGCACAG TTTGAGCCTT GGTTCAACCA GAATGCGGGC 6885
Consensus ACGCGGTGGC GTGGCGGGTA GACATCGCCA CCGGNCAGGT CGANGCNCAG TTTGAGCCNT GGTTCAACCN GAANGCGGGC
M1021 AACATCAGCC GGTTCTGGCA GGAGGGAGAT GTGGTCTACC TAGCGCGCCG TTATCAGCTA AAGGAGAGGG AGTCCGAGCT 6974
TT01 CATATCAGCC GGTTCTGGCA GGAGGGAGAT GTGGTCTACT TGGCGCGCCG TTATCGGCTA AAGGAGAGGG AGGCTGAACT 6965
Consensus NANATCAGCC GGTTCTGGCA GGAGGGAGAT GTGGTCTACN TNGCGCGCCG TTATCNGCTA AAGGAGAGGG AGNCNGANCT
Conservation
Sequence logo

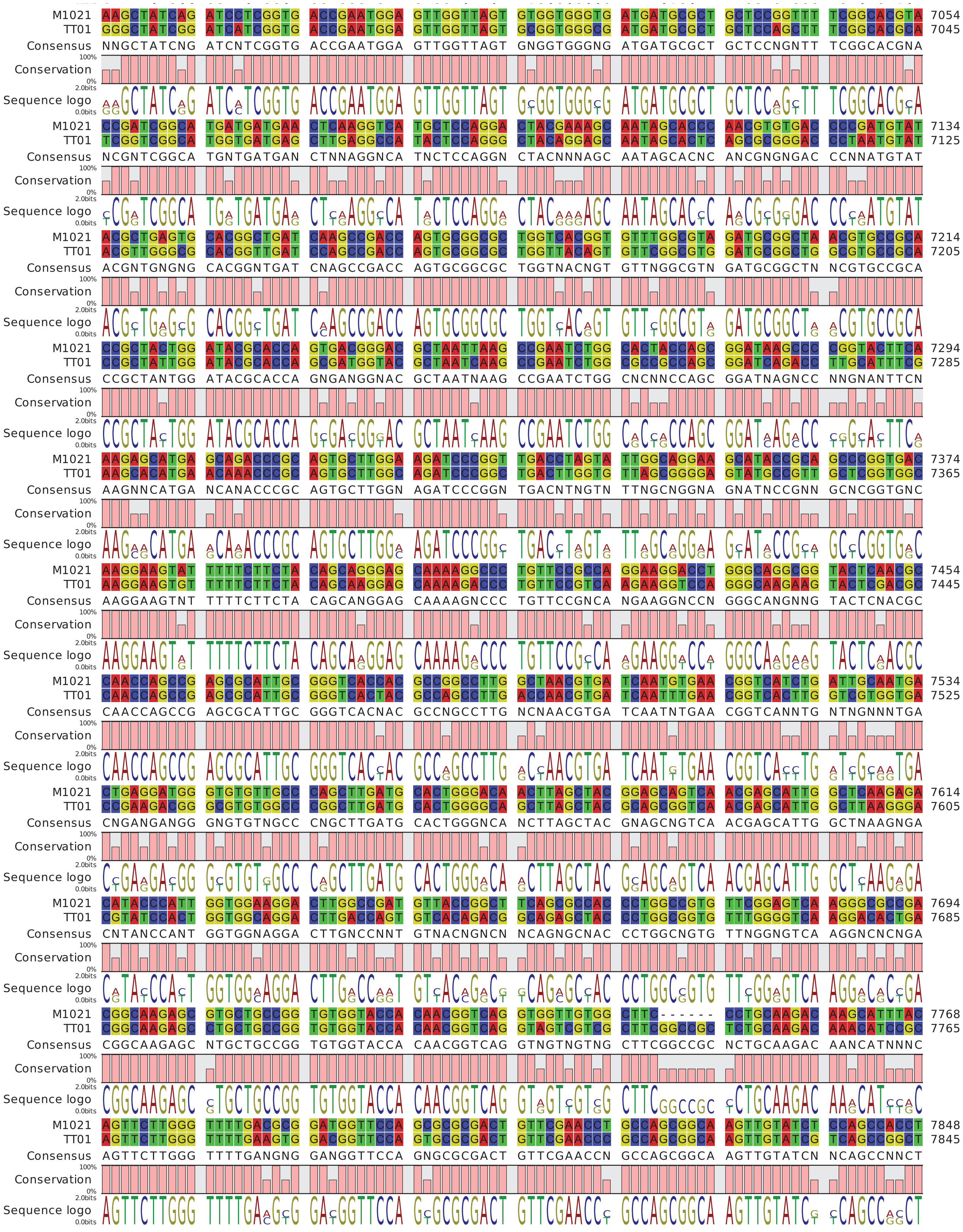
M1021 AAGCTATCAG ATCCTCGGTG ACCGAATGGA GTTGGTTAGT GTGGTGGGTG ATGATGCGCT GCTCCGGTTT TCGGCACGTA 7054
TT01 GGGCTATCGG ATCATCGGTG ACCGAATGGA GTTGGTTAGT GCGGTGGGCG ATGATGCGCT GCTCCAGCTT TCGGCACGCA 7045
Consensus NNGCTATCNG ATCNTCGGTG ACCGAATGGA GTTGGTTAGT GNGGTGGGNG ATGATGCGCT GCTCCNGNTT TCGGCACGNA
M1021 CCGATCGGCA TGATGATGAA CTCAAGGTCA TGCTCCAGGA CTACGAAAGC AATAGCACCC AACGTGTGAC CCCGATGTAT 7134
TT01 TCGGTCGGCA TGGTGATGAG CTTGAGGCCA TACTCCAGGG CTACAGGAGC AATAGCACTC AGCGCGGGAC CCTAATGTAT 7125
Consensus NCGNTCGGCA TGNTGATGAN CTNNAGGNCA TNCTCCAGGN CTACNNNAGC AATAGCACNC ANCGNGNGAC CCNNATGTAT
M1021 ACGCTGAGTG CACGGCTGAT CAAGCCGACC AGTGCGGCGC TGGTCACGGT GTTTGGCGTA GATGCGGCTA ACGTGCCGCA 7214
TT01 ACGTTGGGCG CACGGTTGAT CCAGCCGACC AGTGCGGCGC TGGTTACAGT GTTCGGCGTG GATGCGGCTG GCGTGCCGCA 7205
Consensus ACGNTGNGNG CACGGNTGAT CNAGCCGACC AGTGCGGCGC TGGTNACNGT GTTNGGCGTN GATGCGGCTN NCGTGCCGCA
M1021 CCGCTACTGG ATACGCACCA GTGACGGGAC GCTAATTAAG CCGAATCTGG CACTACCAGC GGATAAGCCC CGGTACTTCA 7294
TT01 CCGCTATTGG ATACGCACCA GCGATGGTAC GCTAATCAAG CCGAATCTGG CGCCGCCAGC GGATCAGACC TTGCATTTCG 7285
Consensus CCGCTANTGG ATACGCACCA GNGANGGNAC GCTAATNAAG CCGAATCTGG CNCNNCCAGC GGATNAGNCC NNGNANTTCN
M1021 AAGAGCATGA GCAGACCCGC AGTGCTTGGA AGATCCCGGT TGACCTAGTA TTGGCAGGAA GCATACCGCA GCCCGGTGAC 7374
TT01 AAGCACATGA ACAAACCCGC AGTGCTTGGC AGATCCCGGC TGACTTGGTG TTAGCGGGGA GTATGCCGTT GCTCGGTGGC 7365
Consensus AAGNNCATGA NCANACCCGC AGTGCTTGGN AGATCCCGGN TGACNTNGTN TTNGCNGGNA GNATNCCGNN GCNCGGTGNC
M1021 AAGGAAGTAT TTTTCTTCTA CAGCAGGGAG CAAAAGGCCC TGTTCCGCCA GGAAGGACCT GGGCAGGCGG TACTCAACGC 7454
TT01 AAGGAAGTGT TTTTCTTCTA CAGCAAGGAG CAAAAGACCC TGTTCCGTCA AGAAGGTCCA GGGCAAGAAG TACTCGACGC 7445
Consensus AAGGAAGTNT TTTTCTTCTA CAGCANGGAG CAAAAGNCCC TGTTCCGNCA NGAAGGNCCN GGGCANGNNG TACTCNACGC
M1021 CAACCAGCCG AGCGCATTGC GGGTCACCAC GCCGGCCTTG GCTAACGTGA TCAATGTGAA CGGTCATCTG ATTGCAATGA 7534
TT01 CAACCAGCCG AGCGCATTGC GGGTCACTAC GCCAGCCTTG ACCAACGTGA TCAATTTGAA CGGTCACTTG GTCGTGGTGA 7525
Consensus CAACCAGCCG AGCGCATTGC GGGTCACNAC GCCNGCCTTG NCNAACGTGA TCAATNTGAA CGGTCANNTG NTNGNNNTGA
M1021 CTGAGGATGG GTGTGTTGCC CAGCTTGATG CACTGGGACA ACTTAGCTAC GGAGCAGTCA ACGAGCATTG GCTCAAGAGA 7614
TT01 CCGAAGACGG GCGTGTGGCC CGGCTTGATG CACTGGGGCA GCTTAGCTAC GCAGCGGTCA ACGAGCATTG GCTTAAGGGA 7605
Consensus CNGANGANGG GNGTGTNGCC CNGCTTGATG CACTGGGNCA NCTTAGCTAC GNAGCNGTCA ACGAGCATTG GCTNAAGNGA
M1021 CATACCCATT GGTGGAAGGA CTTGGCCGAT GTTACCGGCT TCAGCGCCAC CCTGGCCGTG TTCGGAGTCA AGGGCGCCGA 7694
TT01 CGTATCCACT GGTGGCAGGA CTTGACCAGT GTCACAGACG GCAGAGCTAC CCTGGCGGTG TTTGGGGTCA AGGACACTGA 7685
Consensus CNTANCCANT GGTGGNAGGA CTTGNCCNNT GTNACNGNCN NCAGNGCNAC CCTGGCNGTG TTNGGNGTCA AGGNCNCNGA
M1021 CGGCAAGAGC GTGCTGCCGG TGTGGTACCA CAACGGTCAG GTGGTTGTGG CTTC------ CCTGCAAGAC AAGCATTTAC 7768
TT01 CGGCAAGAGC CTGCTGCCGG TGTGGTACCA CAACGGTCAG GTAGTCGTCG CTTCGGCCGC TCTGCAAGAC AAACATCCGC 7765
Consensus CGGCAAGAGC NTGCTGCCGG TGTGGTACCA CAACGGTCAG GTNGTNGTNG CTTCGGCCGC NCTGCAAGAC AANCATNNNC
M1021 AGTTCTTGGG TTTTGACGCG GATGGTTCCA GCGCGCGACT GTTCGAACCT GCCAGCGGCA AGTTGTATCT CCAGCCACCT 7848
TT01 AGTTCTTGGG TTTTGAAGTG GACGGTTCCA GTGCGCGACT GTTCGAACCC GCCAGCGGCA AGTTGTATCG TCAGCCGGCT 7845
Consensus AGTTCTTGGG TTTTGANGNG GANGGTTCCA GNGCGCGACT GTTCGAACCN GCCAGCGGCA AGTTGTATCN NCAGCCNNCT
Conservation
Sequence logo

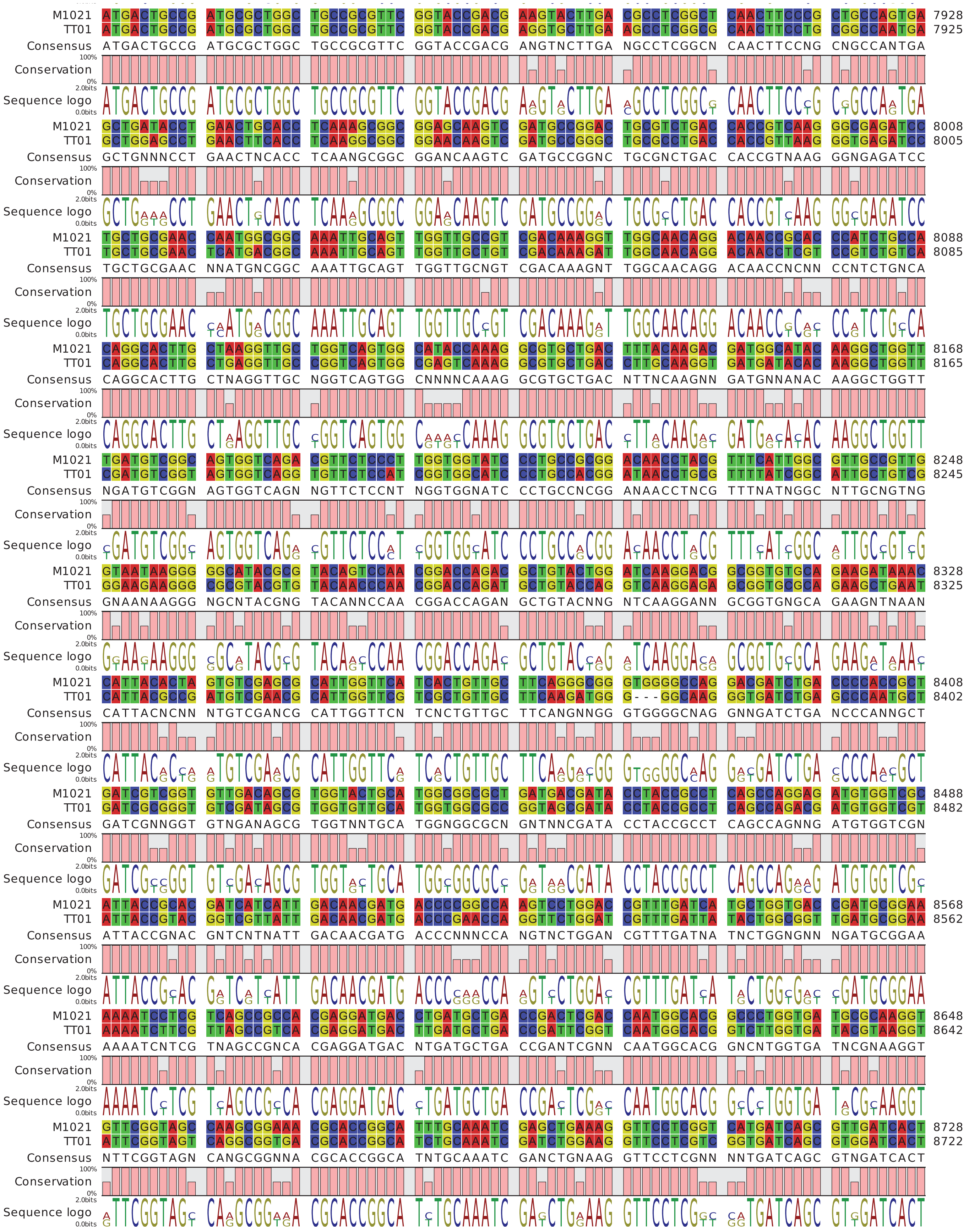
M1021 ATGACTGCCG ATGCGCTGGC TGCCGCGTTC GGTACCGACG AAGTACTTGA CGCCTCGGCT CAACTTCCCG CTGCCAGTGA 7928
TT01 ATGACTGCCG ATGCGCTGGC TGCCGCGTTC GGTACCGACG AGGTGCTTGA AGCCTCGGCG CAACTTCCTG CGGCCAATGA 7925
Consensus ATGACTGCCG ATGCGCTGGC TGCCGCGTTC GGTACCGACG ANGTNCTTGA NGCCTCGGCN CAACTTCCNG CNGCCANTGA
Conservation
Sequence logo
M1021 GCTGATACCT GAACTGCACC TCAAAGCGGC GGAGCAAGTC GATGCCGGAC TGCGTCTGAC CACCGTCAAG GGCGAGATCC 8008
TT01 GCTGGAGCCT GAACTTCACC TCAAGGCGGC GGAACAAGTC GATGCCGGGC TGCGCCTGAC CACCGTTAAG GGTGAGATCC 8005
Consensus GCTGNNNCCT GAACTNCACC TCAANGCGGC GGANCAAGTC GATGCCGGNC TGCGNCTGAC CACCGTNAAG GGNGAGATCC
Conservation
Sequence logo
M1021 TGCTGCGAAC CAATGGCGGC AAATTGCAGT TGGTTGCCGT CGACAAAGGT TGGCAACAGG ACAACCGCAC CCATCTGCCA 8088
TT01 TGCTGCGAAC TCATGACGGC AAATTGCAGT TGGTTGCTGT CGACAAAGAT TGGCAACAGG ACAACCTCGT CCGTCTGTCA 8085
Consensus TGCTGCGAAC NNATGNCGGC AAATTGCAGT TGGTTGCNGT CGACAAAGNT TGGCAACAGG ACAACCNCNN CCNTCTGNCA
Conservation
Sequence logo
M1021 CAGGCACTTG CTAAGGTTGC TGGTCAGTGG CATACCAAAG GCGTGCTGAC TTTACAAGAC GATGGCATAC AAGGCTGGTT 8168
TT01 CAGGCACTTG CTGAGGTTGC CGGTCAGTGG CGAGTCAAAG GCGTGCTGAC CTTGCAAGGT GATGATACAC AAGGCTGGTT 8165
Consensus CAGGCACTTG CTNAGGTTGC NGGTCAGTGG CNNNNCAAAG GCGTGCTGAC NTTNCAAGNN GATGNNANAC AAGGCTGGTT
Conservation
Sequence logo
M1021 TGATGTCGGC AGTGGTCAGA CGTTCTCCCT TGGTGGTATC CCTGCCGCGG ACAACCTACG TTTCATTGGC GTTGCCGTTG 8248
TT01 CGATGTCGGT AGTGGTCAGG TGTTCTCCAT CGGTGGCATC CCTGCCACGG ATAACCTGCG TTTTATCGGC ATTGCTGTCG 8245
Consensus NGATGTCGGN AGTGGTCAGN NGTTCTCCNT NGGTGGNATC CCTGCCNCGG ANAACCTNCG TTTNATNGGC NTTGCNGTNG
Conservation
Sequence logo
M1021 GTAATAAGGG GGCATACGCG TACAGTCCAA CGGACCAGAC GCTGTACTGG ATCAAGGACG GCGGTGTGCA GAAGATAAAC 8328
TT01 GGAAGAAGGG CGCGTACGTG TACAACCCAA CGGACCAGAT GCTGTACCAG GTCAAGGAGA GCGGTGCGCA GAAGCTGAAT 8325
Consensus GNAANAAGGG NGCNTACGNG TACANNCCAA CGGACCAGAN GCTGTACNNG NTCAAGGANN GCGGTGNGCA GAAGNTNAAN
Conservation
Sequence logo
M1021 CATTACACTA GTGTCGAGCG CATTGGTTCA TCACTGTTGC TTCAGGGCGG GTGGGGCCAG GACGATCTGA CCCCACCGCT 8408
TT01 CATTACGCCG ATGTCGAACG CATTGGTTCG TCGCTGTTGC TTCAAGATGG G---GGCAAG GGTGATCTGA GCCCAATGCT 8402
Consensus CATTACNCNN NTGTCGANCG CATTGGTTCN TCNCTGTTGC TTCANGNNGG GTGGGGCNAG GNNGATCTGA NCCCANNGCT
Conservation
Sequence logo
M1021 GATCGTCGGT GTTGACAGCG TGGTACTGCA TGGCGGCGCT GATGACGATA CCTACCGCCT CAGCCAGGAG ATGTGGTCGC 8488
TT01 GATCGCGGGT GTCGATAGCG TGGTGTTGCA TGGTGGCGCC GGTAGCGATA CCTACCGCCT CAGCCAGACG ATGTGGTCGT 8482
Consensus GATCGNNGGT GTNGANAGCG TGGTNNTGCA TGGNGGCGCN GNTNNCGATA CCTACCGCCT CAGCCAGNNG ATGTGGTCGN
Conservation
Sequence logo
M1021 ATTACCGCAC GATCATCATT GACAACGATG ACCCCGGCCA AGTCCTGGAC CGTTTGATCA TGCTGGTGAC CGATGCGGAA 8568
TT01 ATTACCGTAC GGTCGTTATT GACAACGATG ACCCGAACCA GGTTCTGGAT CGTTTGATTA TACTGGCGGT TGATGCGGAA 8562
Consensus ATTACCGNAC GNTCNTNATT GACAACGATG ACCCNNNCCA NGTNCTGGAN CGTTTGATNA TNCTGGNGNN NGATGCGGAA
Conservation
Sequence logo
M1021 AAAATCCTCG TCAGCCGCCA CGAGGATGAC CTGATGCTGA CCGACTCGAC CAATGGCACG GCCCTGGTGA TGCGCAAGGT 8648
TT01 AAAATCTTCG TTAGCCGTCA CGAGGATGAC TTGATGCTGA CCGATTCGGT CAATGGCACG GTCTTGGTGA TACGTAAGGT 8642
Consensus AAAATCNTCG TNAGCCGNCA CGAGGATGAC NTGATGCTGA CCGANTCGNN CAATGGCACG GNCNTGGTGA TNCGNAAGGT
Conservation
Sequence logo
M1021 GTTCGGTAGC CAAGCGGAAA CGCACCGGCA TTTGCAAATC GAGCTGAAAG GTTCCTCGGT CATGATCAGC GTTGATCACT 8728
TT01 ATTCGGTAGT CAGGCGGTGA CGCACCGGCA TCTGCAAATC GATCTGGAAG GTTCCTCGTC GGTGATCAGC GTGGATCACT 8722
Consensus NTTCGGTAGN CANGCGGNNA CGCACCGGCA TNTGCAAATC GANCTGNAAG GTTCCTCGNN NNTGATCAGC GTNGATCACT
Conservation
Sequence logo

S20

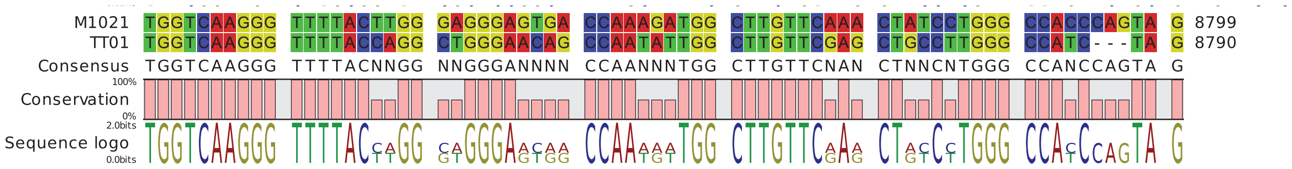
M1021 TGGTCAAGGG TTTTACTTGG GAGGGAGTGA CCAAAGATGG CTTGTTCAAA CTATCCTGGG CCACCCAGTA G 8799
TT01 TGGTCAAGGG TTTTACCAGG CTGGGAACAG CCAATATTGG CTTGTTCGAG CTGCCTTGGG CCATC---TA G 8790
Consensus TGGTCAAGGG TTTTACNNGG NNGGGANNNN CCAANNNTGG CTTGTTCNAN CTNNCNTGGG CCANCCAGTA G
100%
Conservation
0%
2.0bits
Sequence logo
0.0bits

Figure S2. Diagrammatic presentation of domain structure of the Mcf insecticidal toxin from *P. temperata* M1021 and showing the relative location of three potential functional domains in the Mcf protein. (**I**) First, the N-terminus carries a BH3-like consensus motif, suggesting the protein may be pro-apoptotic; (**II**) A centrally located domain with similarity to the translocation domain of *C. difficile* toxin B; (**III**) The C-terminal domain carrying a sequence repeated in the C-terminus of ApxIVA from *Actinobacillus pleuropneumoniae*, an RTX-like toxin.

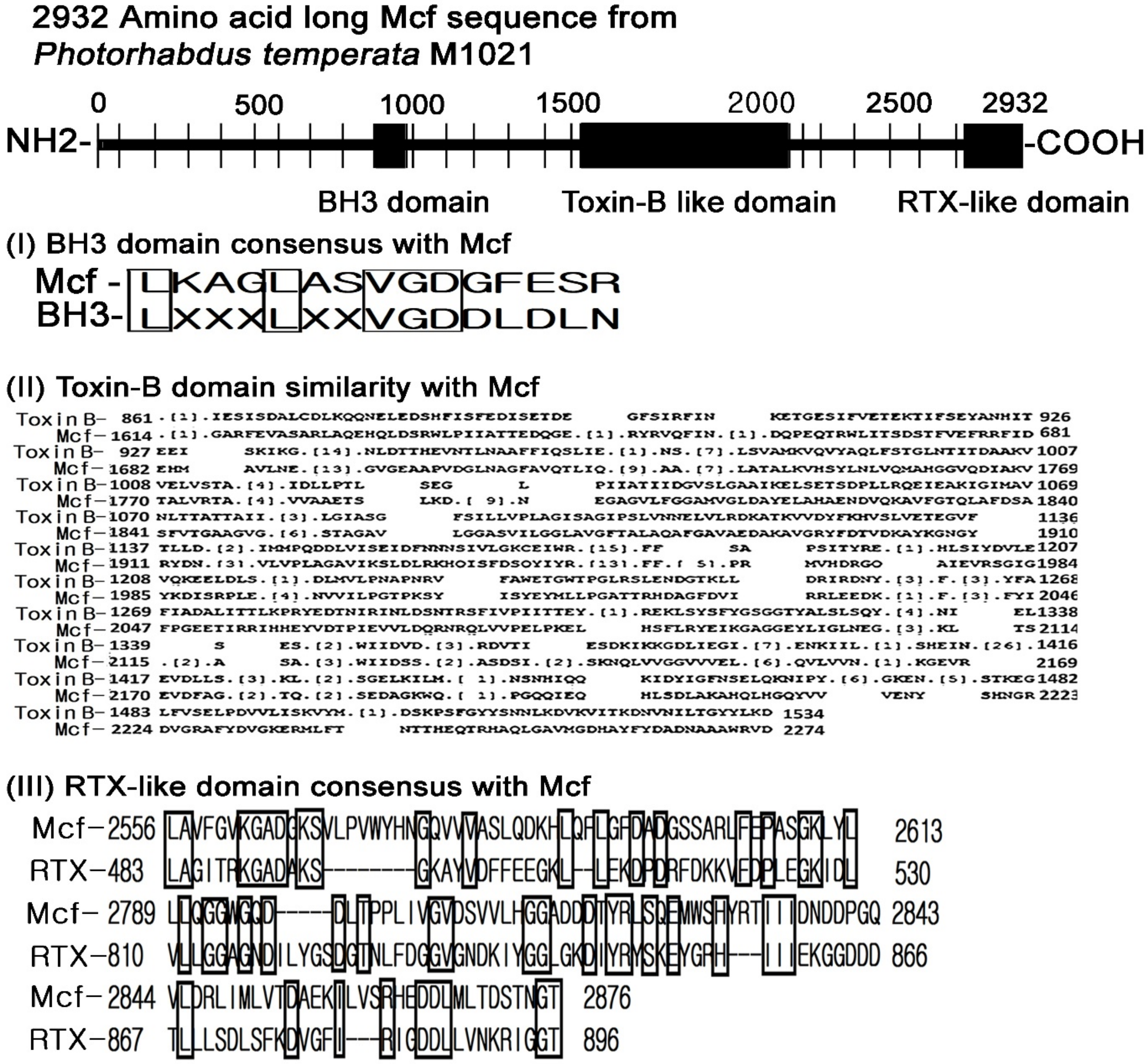

Supplement: Supplementary File 1 — Supplementary Files (PDF, 3182 KB) [file toxins-06-02024-s001.pdf]
